# LACTB exerts tumor suppressor properties in epithelial ovarian cancer through regulation of Slug

**DOI:** 10.26508/lsa.202201510

**Published:** 2022-11-14

**Authors:** Valentina Cutano, Jessica Marianne Ferreira Mendes, Sara Escudeiro-Lopes, Susana Machado, Judith Vinaixa Forner, Juan M Gonzales-Morena, Martin Prevorovsky, Viacheslav Zemlianski, Yuxiong Feng, Petra Kralova Viziova, Andrea Hartmanova, Beata Malcekova, Pavel Jakoube, Sonia Iyer, Zuzana Keckesova

**Affiliations:** 1 Institute of Organic Chemistry and Biochemistry, Czech Academy of Sciences, Prague, Czech Republic; 2 Department of Cell Biology, Faculty of Science, Charles University, Prague, Czech Republic; 3 Zhejiang Provincial Key Laboratory of Pancreatic Disease, First Affiliated Hospital, and Institute of Translational Medicine, Zhejiang University School of Medicine, Hangzhou, China; 4 The Czech Center for Phenogenomics, Institute of Molecular Genetics of the Czech Academy of Sciences, Vestec, Czech Republic; 5 Whitehead Institute for Biomedical Research, Cambridge, MA, USA

## Abstract

Epithelial–mesenchymal transition (EMT) is a cellular mechanism used by cancer cells to acquire migratory and stemness properties. In this study, we show, through in vitro, in vivo, and 3D culture experiments, that the mitochondrial protein LACTB manifests tumor suppressor properties in ovarian cancer. We show that LACTB is significantly down-regulated in epithelial ovarian cancer cells and clinical tissues. Re-expression of LACTB negatively effects the growth of cancer cells but not of non-tumorigenic cells. Mechanistically, we show that LACTB leads to differentiation of ovarian cancer cells and loss of their stemness properties, which is achieved through the inhibition of the EMT program and the LACTB-dependent down-regulation of Snail2/Slug transcription factor. This study uncovers a novel role of LACTB in ovarian cancer and proposes new ways of counteracting the oncogenic EMT program in this model system.

## Abstract (precis)

We show the ability of LACTB to function as a tumor suppressor in ovarian cancer through down-regulation of Slug and induction of differentiation.

## Introduction

Ovarian cancer is one of the major causes of lethal gynecologic malignancies (1). In 2020, there were an estimated 313,959 cases and 207,252 deaths from ovarian cancer worldwide (2). Late diagnosis when patients are in advanced stages is most likely the main cause of the high mortality (3). Around 90% of primary ovarian tumors are of epithelial origin classified as epithelial ovarian cancer which include serous, endometrioid, mucinous, transitional cell, clear cell, mixed epithelial, undifferentiated, and unclassified (1). Moreover, clinical observations and genetic studies have divided ovarian cancers into two major subtypes: type 1 and type 2 ovarian cancers (4). Type 1 ovarian cancers tend to behave in a more indolent way and grow locally, such as low-grade serous or mucinous cancers. Type 2 ovarian cancers are highly aggressive malignancies, such as high-grade serous cancers, most of which were recently described to originate in the fallopian tubes (5). Determination of early diagnosis markers or therapeutic targets to improve the diagnosis and prognosis of ovarian cancer patients is currently the focus of many researchers all over the world.

Epithelial–mesenchymal transition (EMT) is a cellular process in which cells transit from epithelial state to a mesenchymal state (6, 7). As such, during the EMT process, cells acquire new migratory and stemness properties. This cellular program is often hijacked and exploited by cancer cells, what leads to enhancement of their aggressiveness, migration, and metastasis-forming abilities (8, 9). EMT can be induced in cancer cells through various mechanisms, which include aberrant activation of Wnt and Notch signaling pathways, TGFβ, bone morphogenetic proteins, and receptor tyrosine kinases (10). This leads to activation of EMT transcription factors such as Snail, Slug, Twist, and Zeb1/2 to promote the down-regulation of epithelial-related genes such as E-cadherin (E-cad) and, in parallel, enhancement of the expression of mesenchymal-related genes such as vimentin (8, 11, 12). EMT is a critical step in the progression of cancer in many (albeit not all) tissues, including epithelial ovarian cancer (13), where induction of EMT (through up-regulation of Snail, Twist, and Zeb1/2) is associated with poor overall survival of patients (14). Therefore, finding ways to inhibit the EMT process and promote the differentiation in ovarian cancer cells represents a possible therapeutic strategy to improve ovarian cancer patient survival.

The eukaryotic mitochondrial protein serine b-lactamase–like protein (LACTB) evolutionarily belongs to the bacterial penicillin-binding/b-lactamase protein family (15, 16, 17). Recently, Keckesova et al described LACTB as a novel tumor suppressor in breast cancer, showing that LACTB has the ability to change mitochondrial lipid metabolism and, through such reprogramming, to modulate the differentiation state of cancer cells (18). This resulted in decreased cancer cell proliferation and impaired tumorigenesis. Following studies extended the tumor suppressive role of LACTB to different cancer tissues such as colorectal cancer, glioma, gastric cancer, and melanoma (17, 19, 20, 21, 22). Considering the strong tumor suppressive activity of LACTB in cancers of epithelial origin, we decided to investigate the role of LACTB in epithelial ovarian cancer.

Our study showed that LACTB is significantly down-regulated in ovarian cancer cells and in human ovarian cancer tissues. We show that re-expression of LACTB in ovarian cancer negatively affects the growth of different ovarian cell lines in vitro and in vivo. Furthermore, we show that expression of LACTB leads to loss of cancer stem cell properties and induction of differentiation. The mechanism responsible for this tumor suppressive effect is the LACTB-induced inhibition of the EMT program in ovarian cancer cells, which happens through LACTB-dependent down-regulation of Slug EMT transcription factor. As such, this study shows for the first time the tumor suppressive role of LACTB in ovarian cancers and the importance of Slug EMT transcription factor in its mechanism.

## Results

### LACTB is down-regulated in ovarian cancer cells and tissues

**Figure 1.**
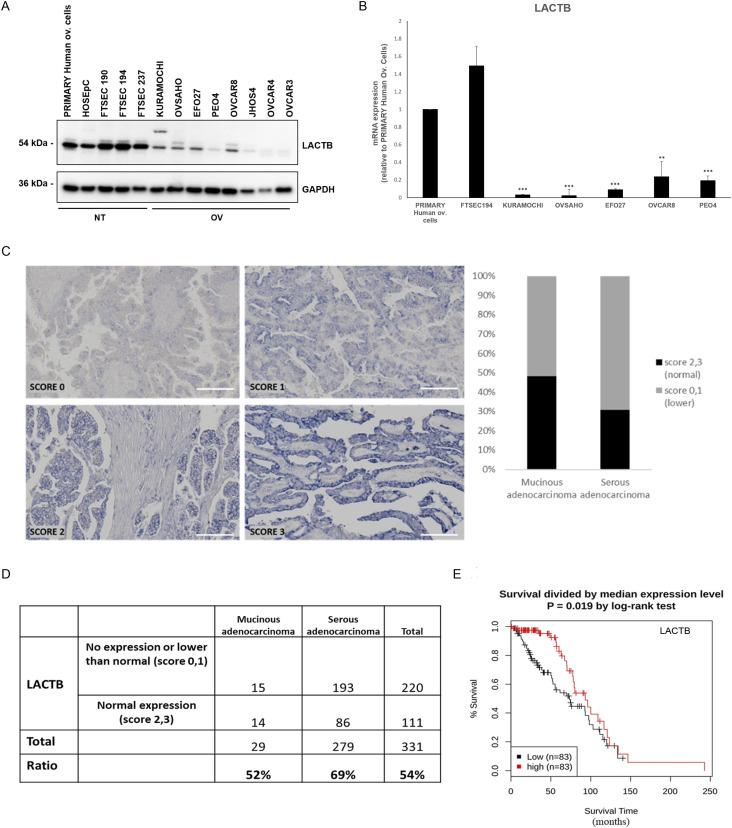
LACTB is down-regulated in ovarian cancer cells and tissues. **(A)** Western blot of endogenous LACTB protein levels in a panel of non-tumorigenic and tumorigenic ovarian cancer cell lines. NT, non-tumorigenic cells; OV, ovarian cancer cell lines. GAPDH was used as a loading control. **(B)** LACTB mRNA expression levels in a panel of ovarian cells were measured by quantitative real-time PCR and indicated as mean ± SD. The experiment was repeated twice. **(C)** Representative immunohistochemistry images of endogenous LACTB protein levels in a panel of clinically defined human epithelial ovarian cancer samples. Scores 2 (50–80% of expression) and 3 (80–100% of expression) represent LACTB staining that was considered normal (not down-regulated), whereas scores 0 (0–20% of expression) and 1 (20–50% of expression) represent LACTB staining that was considered down-regulated. Mucinous adenocarcinoma (n = 29), serous adenocarcinoma (n = 279). Magnification 10×. Scale bar, 50 *µ*m. **(D)** Quantification of immunohistochemistry results for LACTB expression level in epithelial ovarian cancer. **(E)** Effects of the expression of LACTB on overall survival in patients with ovarian cancer. Kaplan–Meier curves and log-rank test results derived for overall survival with respect to the expression of LACTB gene. Significance was reached when *P* < 0.05.

**Figure S1.**
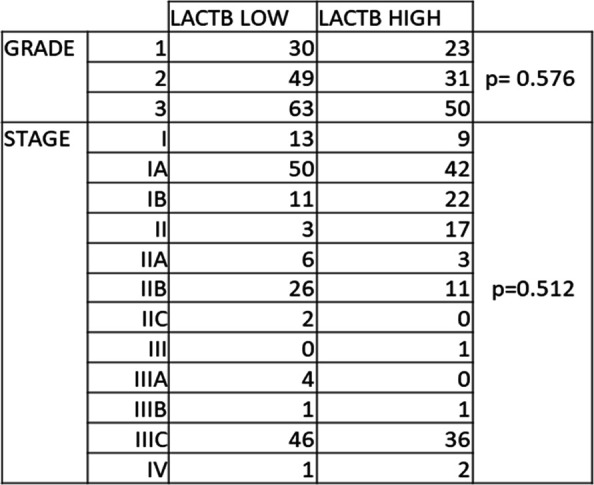
LACTB expression doesn't correlate with tumour. ALAL Stratification of low and high levels of LACTB in ovarian cancer clinical samples of different grades and stages (paired *t* test analysis).

Aiming to investigate the role of LACTB in epithelial ovarian cancer, we first examined its expression in a cohort of ovarian tumorigenic and non-tumorigenic cell lines. Our results show a robust down-regulation of LACTB in ovarian cancer cells compared with non-tumorigenic cell lines (primary human ovarian cells and fallopian tube cells) (Fig 1A). To determine whether the down-regulation of LACTB occurs at transcriptional or post-transcriptional level, we performed RT–PCR analysis which showed significant down-regulation of LACTB mRNA levels in all the tested ovarian cancer cell lines compared with the non-tumorigenic cells used as control (Fig 1B). These results showed that LACTB is down-regulated in ovarian cancer cells, and this down-regulation already occurs at transcriptional level. We next decided to investigate the expression levels of LACTB in 331 clinically defined human ovarian cancer samples to examine our previous findings in human clinical tissues. Because LACTB was shown previously to exert tumor suppressor properties in cancers of epithelial origin, we focused our attention on analyzing the two types of ovary adenocarcinomas: mucinous and serous, which belong to type I and type II of ovarian cancers, respectively. We applied a score (from 0 to 3) based on the intensity of LACTB protein expression compared with the normal tissues. Score 3 represents tissues in which the LACTB expression corresponds to 80–100% of expression intensity in normal tissues, score 2 indicates 50–80%, score 1 indicates 20–50%, and score 0 indicates 0–20% of expression (Fig 1C). In our analysis on tissues, LACTB expression was significantly down-regulated in 52% of mucinous adenocarcinomas and 69% of serous adenocarcinomas when compared with LACTB levels in normal ovary epithelial tissues (n = 26) (Fig 1D). This down-regulation did not correlate with any particular tumor grade or stage (Fig S1). To elucidate how LACTB is clinically relevant to patients with ovarian cancer, we further analyzed the prognostic value of LACTB expression in patients with ovarian cancer. The analysis revealed that patients with high expression levels of LACTB had longer overall survival and better prognosis (*P* = 0.019) (Fig 1E). These data further suggest an important role of LACTB in the management of the malignant effects of ovarian cancer.

### LACTB negatively affects the growth of ovarian cancer cells in vitro

**Figure 2.**
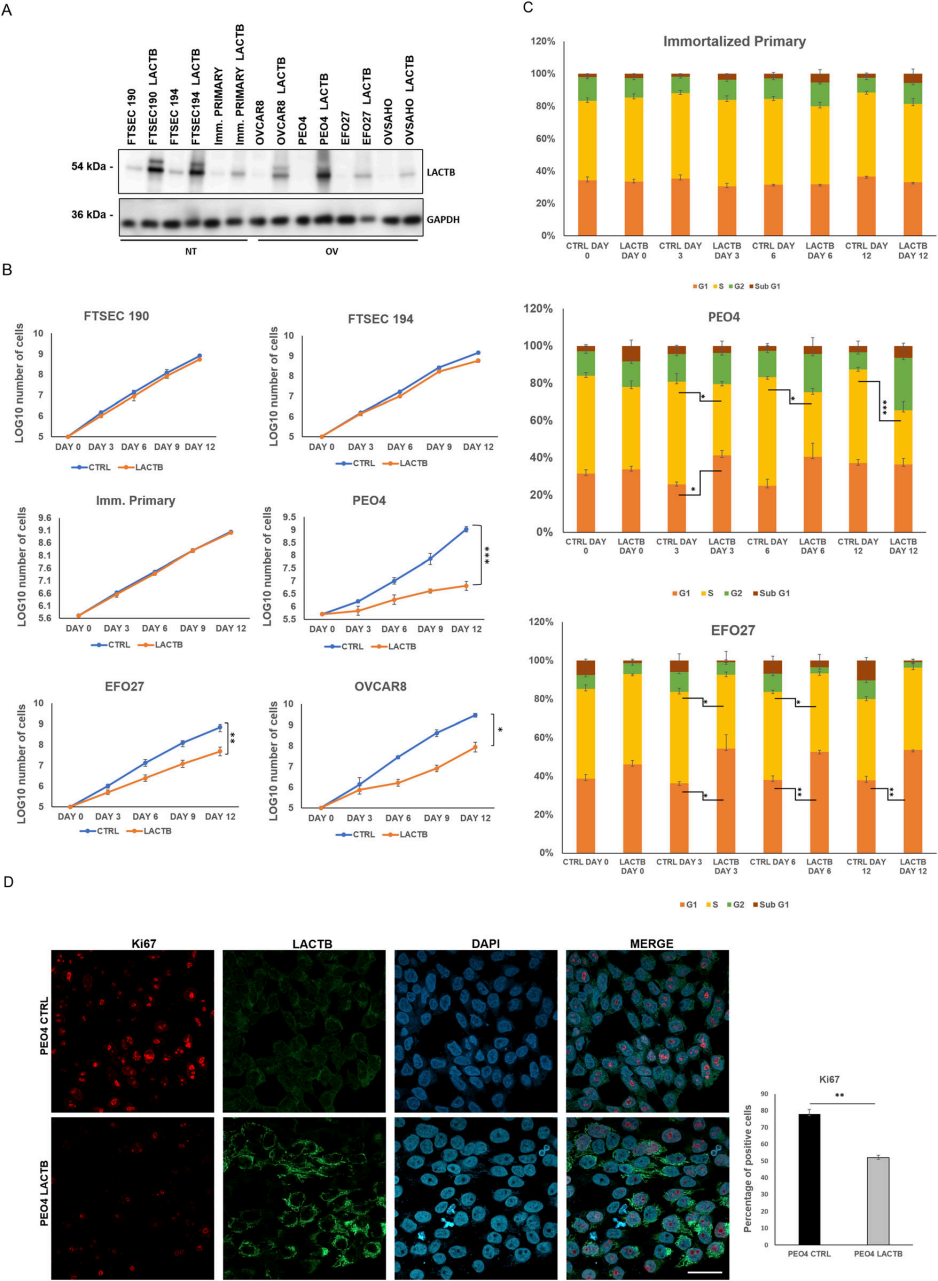
LACTB induction negatively affects the proliferation of ovarian cancer cells. **(A)** Western blot of DOX-inducible LACTB protein levels in a panel of non-tumorigenic and tumorigenic ovarian cancer cell lines. DOX (1 *µ*g/ml) was added to all cells for 6 d. NT, non-tumorigenic cells; OV, ovarian cancer cell lines. GAPDH was used as loading control. **(B)** Proliferation assay of non-tumorigenic and cancer cell lines upon LACTB induction. DOX (1 *µ*g/ml) was added to all cells. Experiments were performed twice. **(C)** Comparison of the DNA synthesis (S-phase) in non-tumorigenic and tumorigenic cell lines upon LACTB induction (day 0, 3, 6, 12). Experiments were performed twice. Significance was reached when *P* < 0.05. **(D)** KI67 immunofluorescence staining and quantification in PEO4 (CTRL and LACTB) cells after 3 d of LACTB induction. Magnification (63×). Scale bar 50 *µ*m. Significance was reached when *P* < 0.05. Experiments were performed twice.

**Figure S2.**
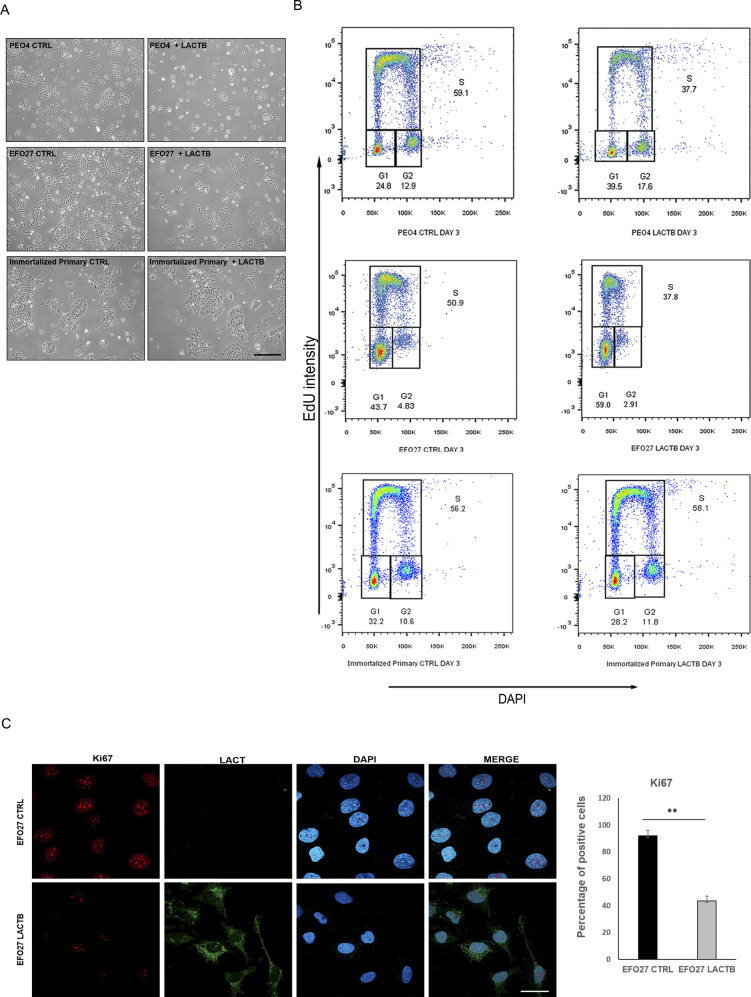
LACTB expression affects ovarian cancer cell growth. **(A)** Representative images of EFO27 and PEO4 upon LACTB induction (6 d). Immortalized primary cells were used as a control. **(B)** EdU staining in cancer cells and immortalized primary cells after 3 d of LACTB induction. **(C)** KI67 immunofluorescence staining and quantification in PEO4 (CTRL and LACTB) cells after 3 d of LACTB induction. Magnification 63×. Scale bar 50 *µ*m. Significance was reached when *P* < 0.05. Experiments were performed twice.

To further understand the role of LACTB in ovarian cancer, we re-introduced a doxycycline-inducible LACTB to ovarian cancer cell lines, which, as we have shown previously, had the endogenous LACTB down-regulated (Fig 2A). Through proliferation experiments, we tested the influence of LACTB in cancer cell lines and non-tumorigenic cell lines (immortalized primary cells and FTSECs) up to 12 d of induction of LACTB. The expression of LACTB in cancer cells significantly reduced their proliferation rate, whereas it had minimal effect on the growth of non-tumorigenic cells (Figs 2B and S2A). This effect was further confirmed by FACS-based EdU assay, which measures the cellular proliferation rate, where we induced LACTB for different time points (0, 3, 6, and 12 d). The result showed a decrease in the percentage of cancer cells in S-phase upon LACTB induction when compared with the immortalized primary cell line used as control (Figs 2C and S2B). Moreover, concomitantly, there was also a significant increase in cancer cells in G1-phase upon LACTB induction, indicative of their cell cycle arrest. We did not detect any significant differences in the sub-G1-phase upon LACTB induction in any of the tested cell lines, showing that LACTB is not promoting apoptosis in these cells. These observations were further confirmed by immunofluorescence staining with the proliferation marker Ki67. PEO4 and EFO27 cells, where LACTB was induced, showed markedly decreased levels of the Ki67 expression than control cell lines without LACTB induction (Figs 2D and S2C). These results showed that the re-expression of LACTB inhibits the proliferation of ovarian cancer cells in vitro and inhibits their progression in the cell cycle.

### LACTB negatively affects the growth of ovarian tumors in vivo

**Figure 3.**
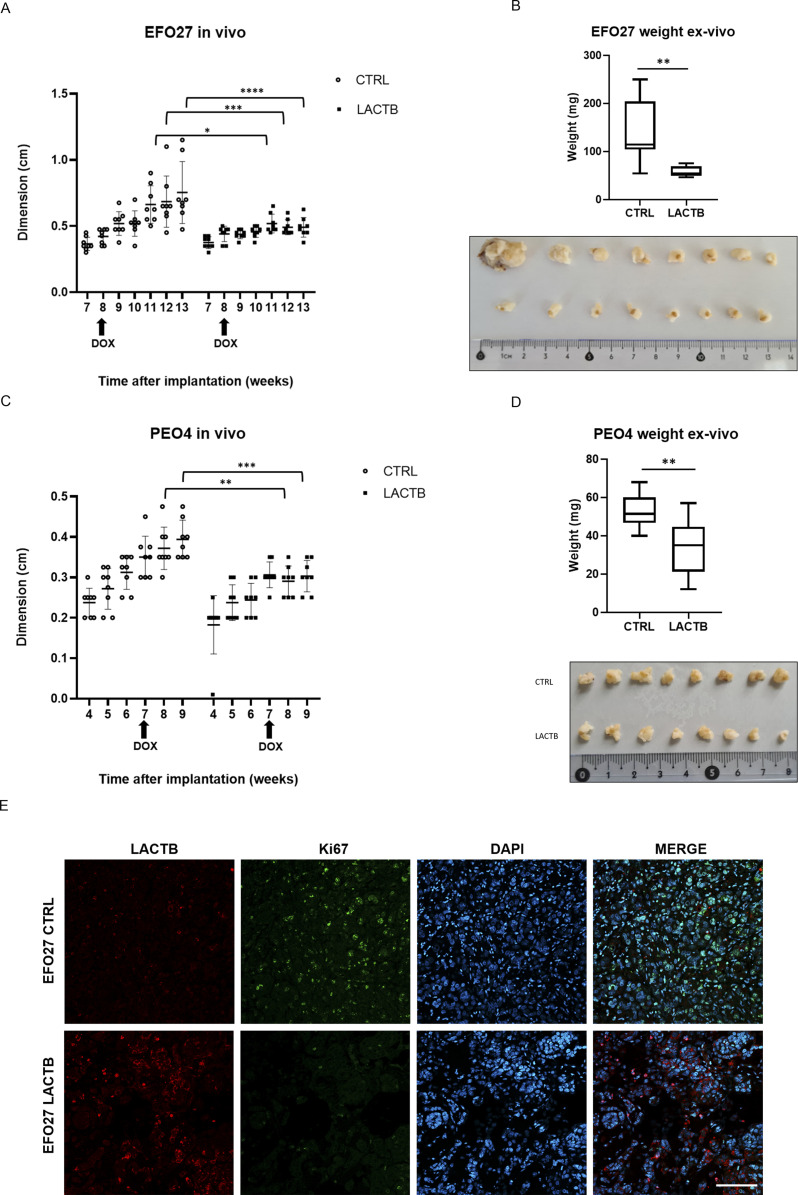
LACTB negatively affects growth of tumors. **(A)** Tumor growth rate upon LACTB induction in EFO27 cells monitored in vivo by tumor measurements. DOX, initiation of doxycycline treatment at the indicated time. Data are mean ± s.e.m. ****P < 0.0001. **(B)** Tumor weight and representative image at the endpoint experiment from EFO27 cells were measured ex vivo at the termination of the experiment. **(C)** Tumor growth rate upon LACTB induction in PEO4 cells monitored in vivo by tumor measurements. DOX, initiation of doxycycline treatment at the indicated time. Data are mean ± s.e.m. ***P < 0.005. **(D)** Tumor weight and representative image at the endpoint experiment from PEO4 cells were measured ex vivo at the termination of the experiment. **(E)** Immunofluorescent staining for the expression of Ki67 in EFO27 CTRL tissue and EFO27 LACTB tissue (20× magnification). Scale bar 50 *µ*m.

We wanted to examine whether the negative effects of LACTB on the proliferation of ovarian cancer cells impact the tumor growth also under in vivo conditions. EFO27 CTRL and EFO27 LACTB cells were orthotopically injected in mice ovaries. Once tumors were formed, LACTB expression was induced through addition of doxycycline to mice, and the growth of tumors was monitored and measured for 4 wk after the treatment initiation. Induction of LACTB significantly inhibited the growth of ovarian tumors within 2–4 wk of LACTB induction, and the tumors expressing LACTB had smaller weight compared with the control tumors (Fig 3A and B). We next extended this finding to another ovarian cancer cell model, PEO4 cells. As previously described, also in this model system, we observed a negative effect of LACTB induction on the growth of ovarian tumors and their weight (Fig 3C and D). These results were further confirmed by immunofluorescence staining in tumor tissues, which showed a marked decrease of Ki67 proliferation marker in tumors with LACTB expression as compared with control tumors (Fig 3E). From these experiments, we can conclude that the expression of LACTB in ovarian tumors contributes to the significant reduction of the tumor size and weight under in vivo conditions. Because both of the cell lines (EFO27 and PEO4) used for the in vivo experiments were derived from metastatic sites, it is reasonable to believe that LACTB re-activation in ovarian cancer metastasis might have a therapeutic effect in counteracting this malignancy.

### LACTB induction leads to decrease in cancer cell stemness properties

**Figure 4.**
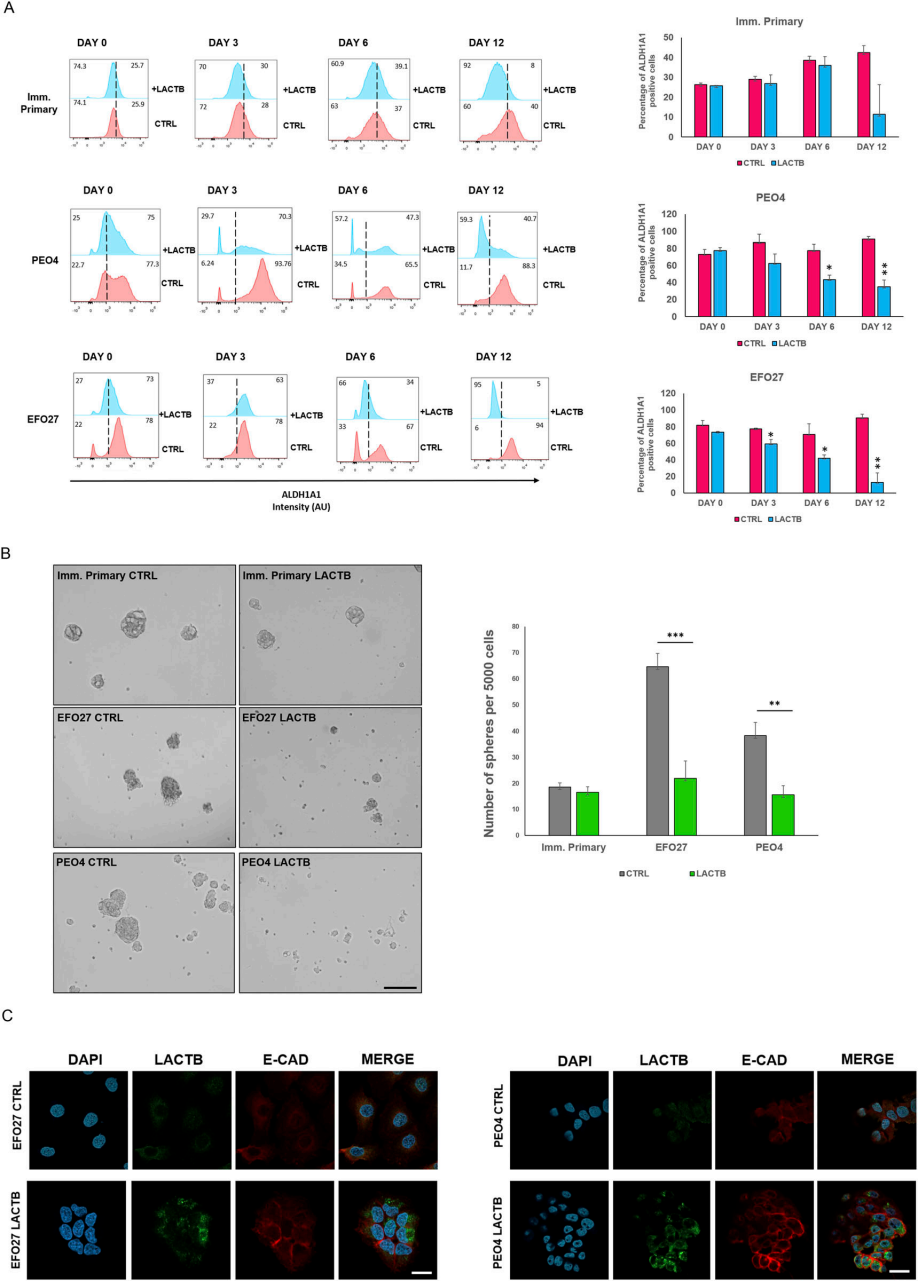
LACTB induction affects stemness properties in ovarian cancer cells. **(A)** Representative flow cytometry analysis and quantification of the ovary stem cell marker ALDH1A in immortalized primary, EFO27, and PEO4 cells upon LACTB induction for 0, 3, 6, and 12 d. Numbers within the graphs represent percentages of gated cells; the line indicates the gate. Significance was reached when *P* < 0.05. Experiments were performed twice. **(B)** Representative sphere images and quantification at day 10 of LACTB induction. EFO27, PEO4, and primary cells were seeded in ultra-low adhesion conditions for 10 d with constant DOX treatment to induce LACTB. Significance was reached when *P* < 0.05. Experiments were performed twice. Magnification (10×). Scale bar 50 *µ*m. **(C)** Immunofluorescence analysis of E-cad expression after 6 d of LACTB induction in EFO27 and PEO4 cells. Magnification 63×. Scale bar 50 *µ*m.

**Figure S3.**
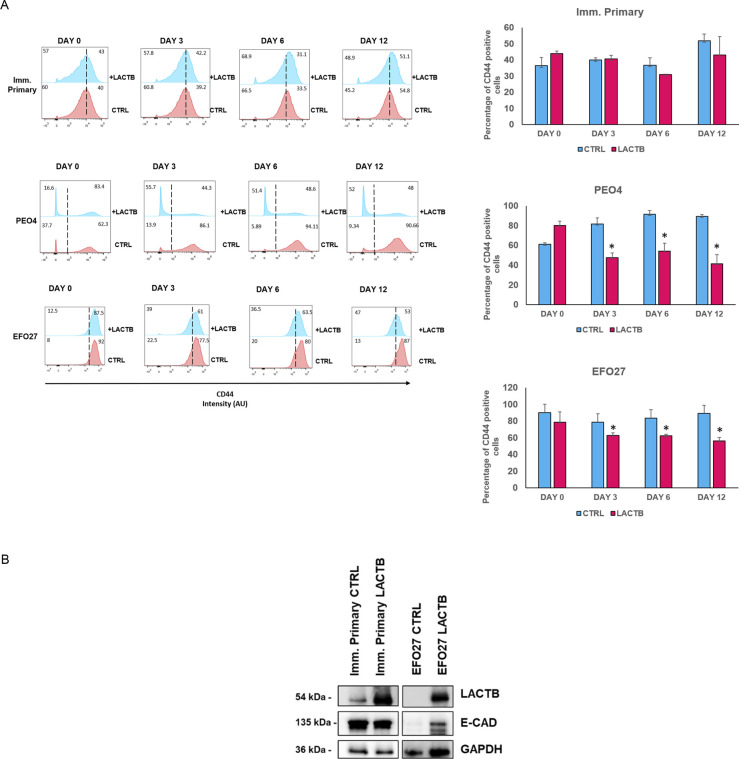
Stem markers are downregulated after LACTB induction in cancer cells. **(A)** Representative flow cytometry analysis and quantification of the ovary stem cell marker ALDH1A in immortalized primary, EFO27, and PEO4 cells upon LACTB induction for 0, 3, 6, and 12 d. Numbers within the graphs represent percentages of gated cells; the dashed line indicates the gate. Significance was reached when *P* < 0.05. Experiments were performed twice. **(B)** Western blot analysis of E-cad expression after LACTB induction in non-tumorigenic (imm. primary) and tumorigenic (EFO 27) cell lines. DOX (1 *µ*g/ml) was added to all cells for 6 d. GAPDH was used as loading control.

Because our previous work in breast cancer model showed that LACTB modulates the stemness of breast cancer cells (18), we decided to examine the impact of LACTB expression on ovarian cancer stem cell properties. We performed an FACS analysis of the commonly used stem cell markers in ovarian cancer (ALDH1A1 and CD44) (11, 23). For this analysis, we selected three cell lines, EFO27 and PEO4 among the cancer cell lines and the immortalized primary ovarian cell line, where we analyzed the expression levels of ALDH1A1 and CD44 after LACTB induction at day 0, 3, 6, and 12 (Figs 4A and S3A). Both cancer cell lines demonstrated decreased expression of both stem markers upon LACTB expression when compared with the control cells. We did not detect any significant difference in the expression of stem markers in the immortalized primary cells, suggesting that the negative effect of LACTB on stem cell population is mostly observed in the tumorigenic state of cells and not in the non-tumorigenic state. To extend these observations to a more physiological 3D culture environment, we investigated the ability of cancer cells to form spheres in the presence or absence of LACTB in a cohort of ovarian cancer and non-tumorigenic cells. Under these conditions, the cells are cultivated in ultra-low adhesion conditions that prevent the growth of differentiated cells and promote the growth of cancer stem cells. The results of this experiment demonstrated a significant negative impact of LACTB expression on the stem properties of the tested ovary cancer cell spheres (Fig 4B). In ovarian cancer cells, E-cadherin loss is frequently associated with a cell matrix adhesion problem, which promotes metastatic behavior (24), and an increase in cancer cell stemness (25, 26). We decided to investigate E-cad expression in cancer cell lines EFO27 CTRL and PEO4 CTRL and in our non-tumorigenic immortalized cells with and without induction of LACTB for 6 d. The immunofluorescence and Western blot analysis showed marked increase in the E-cad expression upon LACTB induction in ovarian cancer cells but not in the non-tumorigenic cells (Figs 4C and S3B). Overall, these results showed that induction of LACTB in ovarian cancer cells leads to decrease in cancer stem cells properties and increase in E-cad. This differentiation-inducing effect of LACTB is manifested mostly in tumorigenic background.

### LACTB induction inhibits the EMT in ovarian cancer cells

**Figure 5.**
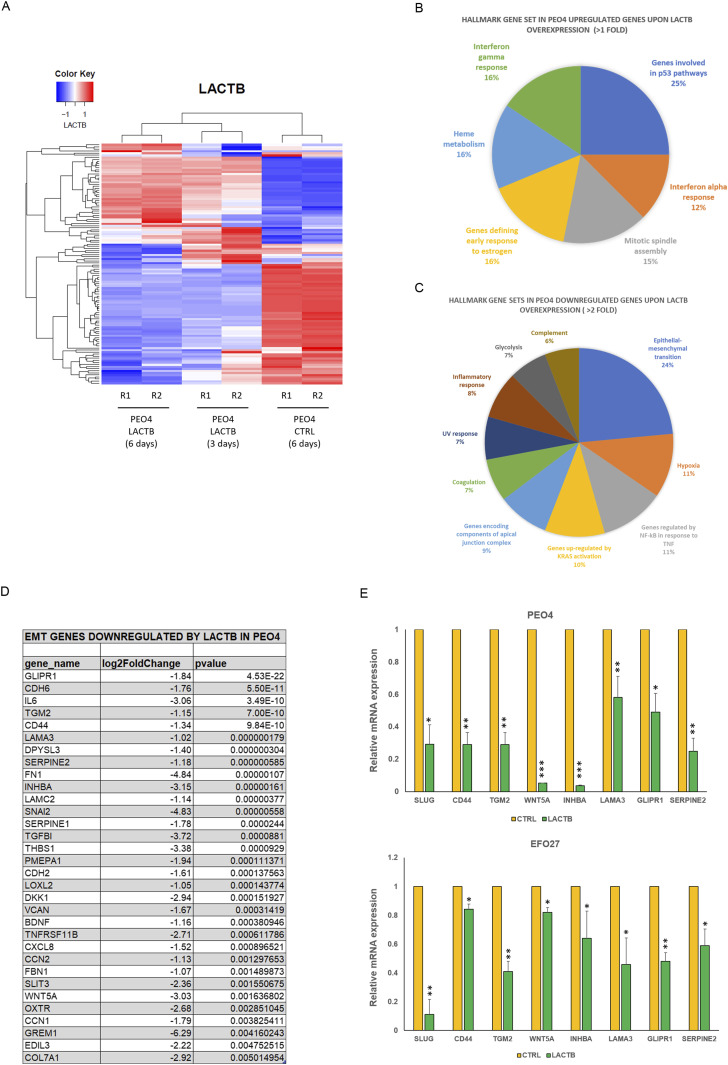
LACTB induction inhibits the EMT program in ovarian cancer cells. **(A)** RNA-seq heat map showing transcriptional changes upon LACTB induction. PEO4 cells were treated with doxycycline treatment (DOX) to induce LACTB expression for 3 and 6 d “PEO4 control (6 d)” refers to cells transfected with empty vector (no LACTB) treated with DOX for 6 d. Gene expression is color-coded with blue and red, designating down-regulation and up-regulation, respectively, and is represented by Z-score. Sample clustering displays the degree of similarity of gene expression among samples (Euclidean distance method). Analysis was performed in duplicates (R1, R2). **(B, C)** Pie charts representing GSEA for genes up-regulated and repressed by LACTB induction in PEO4 when compared with non-tumorigenic immortalized cells at 6 d of induction using MSigDB and hallmark gene sets. **(D)** List of EMT genes down-regulated by LACTB expression in PEO4 cells listed by the *P*-value. **(E)** RNA-seq validation through quantitative real-time PCR in EFO27 and PEO4 cells. Data are represented as mean ± SD. The experiment was repeated twice.

**Figure S4.**
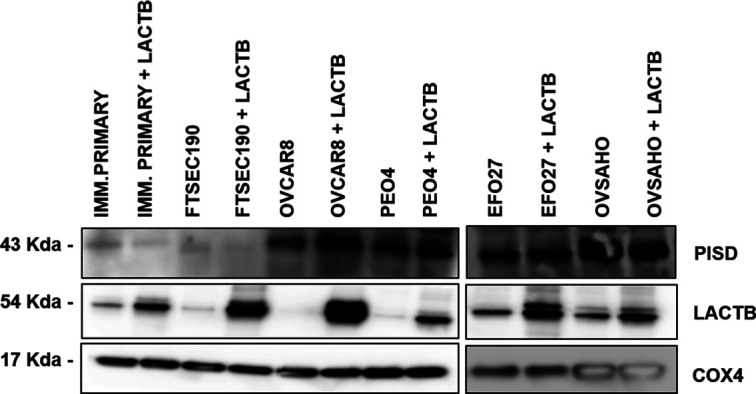
PISD expression is not influenced by the LACTB presence in ovarian cells. Western blot analysis of PISD expression after LACTB induction in the mitochondrial fraction of non-tumorigenic (immortalized primary and FTSEC190) and tumorigenic (OVCAR8, PEO4, EFO27, OVSAHO) cell lines. DOX (1 *µ*g/ml) was added to all cells for 3 d. COX4 was used as loading control.

To map the genetic programs influenced by the expression of LACTB in ovarian cancer, we employed RNA-seq analysis to compare the transcriptomes of non-tumorigenic immortalized cells and PEO4 cells where LACTB was induced for 3 and 6 d (Fig 5A–C). Fig 5B and C depict the gene families that are differentially expressed in PEO4 cells when compared with non-tumorigenic immortalized cells upon 6 d of LACTB induction. We selected the top differentially expressed genes to generate the heat map (statistical significance *P* < 0.05 and log_2_ [fold change] > 0.5). LACTB-induced expression for 3 d resulted in the significant up-regulation of 53 genes and down-regulation of 63 genes, and LACTB-induced expression for 6 d resulted in the significant up-regulation of 46 genes and down-regulation of 70 genes. Under both conditions, LACTB was the most up-regulated gene, thus validating our approach. We decided to group the families of genes affected by the LACTB expression, and among the most up-regulated genes, we found genes related to the p53 pathway, interferon α response, and mitotic spindle (Fig 5B). Conversely, the families of genes that showed the strongest down-regulation upon LACTB induction were involved in the EMT, hypoxia, and NF-kB response (Fig 5C and D). Considering the important impact of EMT in ovarian cancer progression and the effects of LACTB on ovarian cancer cell stemness presented above, we focused our attention on the EMT gene family to examine its role in LACTB mechanism. We confirmed the RNA-seq results by RT–PCR in two different cell lines (PEO4 and EFO27), which showed a common strong down-regulation of different EMT-related factors (SLUG, CD44, TGM2, WNT5A, INHBA, LAMA3, GLIPR1, SERPINE2) upon LACTB induction for 3 d (Fig 5E). LACTB was previously shown to be associated with lipids and/or lipid metabolism (27 *Preprint*, 28, 29, 30, 31, 32). This was supported by our own results where we showed that in breast cancer model, LACTB exerts its tumor suppressive effect through down-regulation of mitochondrial lipid–synthesizing enzyme phosphatidyl serine decarboxylase (PISD) which is partially responsible for the resulting breast cancer cell differentiation (18). Therefore, we examined whether LACTB induction leads to down-regulation of PISD also in the ovarian cancer background. Fig S4 shows that we did not observe any down-regulation of PISD upon LACTB expression. Moreover, our RNA-seq analysis did not reveal any major changes in pathways involved in lipid metabolism upon LACTB induction. These results showed that in contrast to breast cancer cells, LACTB’s differentiation-inducing potential is, in the context of ovary cancer models, manifested through induction of EMT instead of influencing the PISD enzyme and lipid metabolism.

### LACTB expression decreases Slug/Snail2 levels and interferes with the cellular migration

**Figure 6.**
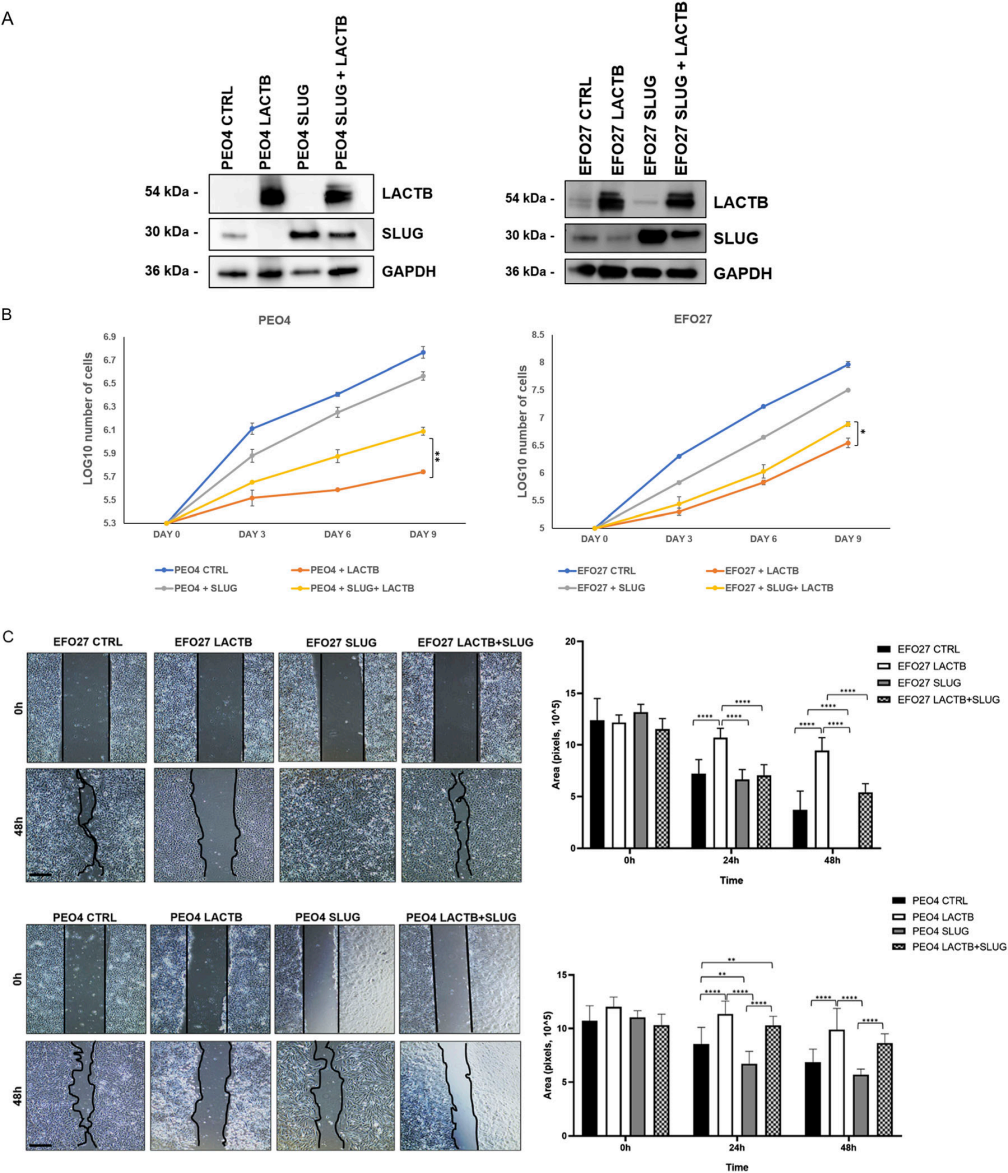
Effect of SLUG/SNAIL2 on LACTB’s tumor suppressive ability. **(A)** Western blot analysis of DOX-inducible LACTB and Slug proteins in EFO27 AND PEO4 cancer cell lines. DOX (1 *µ*g/ml) was added to all cells for 3 d. GAPDH was used as loading control. **(B)** Proliferation assay of cancer cell lines upon LACTB and Slug induction. Experiments were performed twice. **(C)** Migration assays were performed for 48 h after mitomycin treatment in PEO4 and EFO27 cells. After 2 h of mitomycin treatment, DOX (1 *µ*g/ml) was added to all the cells. The experiment was performed twice. Source data are available for this figure.

**Figure S5.**
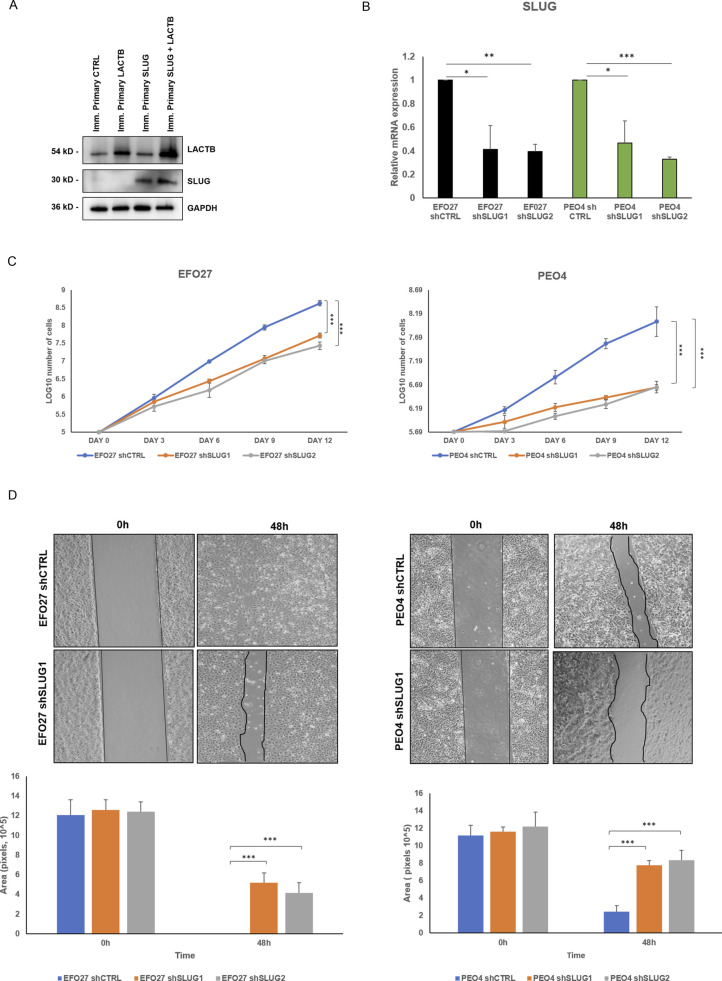
SLUG influences cell growth and migration of ovarian cancer cells. **(A)** Western blot analysis of SLUG expression after LACTB induction in non-tumorigenic cell lines. DOX (1 *µ*g/ml) was added to all cells for 3 d. GAPDH was used as loading control. **(B)** SLUG mRNA expression levels in a panel of ovarian cells were measured by quantitative real-time PCR and indicated as mean ± SD. The experiment was repeated three times. **(C)** Proliferation assay of cancer cell lines upon Slug down-regulation. Experiments were performed three times and indicated as mean ± SD. **(D)** Migration assays were performed for 48 h after mitomycin treatment in PEO4 and EFO27 cells. The experiment was performed three times.

Slug/Snail2 is considered one of the main players of the EMT program in different cancer tissues, including ovarian cancer cells (33, 34, 35, 36). It is known for its capacity to promote cell migration, thus allowing the cancer cells to metastasize (33, 36). Because our results showed that one of the genes with the strongest down-regulation upon LACTB induction was the EMT transcription factor Slug/Snail2, we decided to characterize the role of this protein in the LACTB mechanism in more detail. We expressed doxycycline-inducible Slug in two cancer cell lines (EFO27 and PEO4) and performed the Western blot analysis to examine its protein levels in the presence and absence of LACTB. Slug levels were significantly decreased in both cell lines where LACTB was expressed (Fig 6A). In contrast to cancer cells, LACTB expression does not lead to Slug down-regulation in non-tumorigenic cells (Fig S5A). Moreover, the levels of the endogenous Slug were also decreased in EFO27 cells upon LACTB induction, confirming the results from our RNA-seq analysis. Even though the LACTB induction decreases the protein levels of exogenous, doxycycline-inducible Slug, the overall Slug levels are higher than those of the endogenous Slug expression in both cell lines with LACTB expression. Therefore, we wanted to examine whether the increased Slug levels can counteract the tumor suppressive activity of LACTB. We performed a proliferation experiment where we monitored the growth of two ovarian cancer cell lines (PEO4 and EFO27) in the presence and absence of Slug and LACTB for 9 d. The results showed that increased Slug levels indeed significantly counteracted the tumor suppressive ability of LACTB (Fig 6B). The fact that this effect was partial can be explained by the fact that LACTB is actively down-regulating the Slug expression levels, thus making it difficult for Slug to reach the expression levels that would be high enough to overcome LACTB’s negative effect on cancer cell growth to the full extent. These experiments confirmed that LACTB’s negative effects on the growth of ovarian cancer cells are mostly realized through down-regulation of the Slug EMT transcription factor. Considering the well-known impact of Slug in migration of ovarian cancer cells (36), we decided to monitor the cell migration of two ovarian cancer cell lines with LACTB and Slug expression. The results showed that cells expressing Slug indeed manifested a strong migration potential (Fig 6C). On the other hand, cells where LACTB was expressed had diminished ability to migrate compared with control cells, showing the ability of LACTB to counteract ovarian cancer cell migratory properties (Fig 6C). The negative effect of LACTB on ovarian cancer cell migration was partially reverted by concomitant Slug over-expression, further confirming the important role of Slug in LACTB tumor suppressive mechanism (Fig 6C). Furthermore, we performed down-regulation studies of Slug (shSLUG1 and shSLUG2) in two different ovary cancer cell lines (EFO27 and PEO4). We showed that Slug down-regulation led to impairment of cancer cell proliferation and migration capacities, thus mimicking the effect of LACTB induction on cancer cells (Fig S5B–D).

## Discussion

Ovarian cancer is one of the most lethal types of cancers, and activation of the EMT program was shown to highly increase the aggressivity and metastatic ability of this disease (13, 37, 38). As such, factors promoting EMT in ovarian cancer cells lead to ovarian cancer progression, whereas factors suppressing EMT usually hinder cancer progression. For example, mucin 4 that induces EMT in ovarian cancer cells strongly fosters cancer progression and is often overexpressed in high-grade ovarian tumors (39). On the other hand, the microRNA-200c that deters EMT inhibits metastasis of CD117^+^CD44^+^ ovarian cancer stem cells (37). TGFβ is another example of a protein that can induce the expression of several EMT-TFs through the activation of the SMAD or non-SMAD signaling pathways, which leads to up-regulation of proteins such as SNAIL1, SNAIL2 (Slug), ZEB1/2, and TWIST (40, 41, 42). Among the EMT transcription factors, SNAIL1 and 2 play an important role in ovarian cancer progression because they promote changes in cell–cell junctions and enhancement of invasion properties (36). In particular, Slug/Snail2 promotes EMT in ovarian cancer, increasing the invasiveness of cancer cells (36, 43), and its expression is significantly associated with tumor grade and lymph node metastasis, showing a worse prognosis in patients (33). Therefore, the regulation of the EMT process in ovarian cancer cells represents a target for future therapies with the aim to revert their mesenchymal phenotype into a more differentiated, less tumorigenic state.

Our present study identified new options for halting the EMT process in ovarian cancers. We show that expression of LACTB presents one such option, thus identifying its role as an EMT-inhibiting tumor suppressor in ovarian cancers. We show that expression of LACTB leads to inhibition of ovarian cancer cell growth in vivo and in vitro, decreases cancer stemness properties and migration, and increases ovary cancer cell differentiation. Mechanistically, this occurs through LACTB-dependent down-regulation of Slug transcription factor. In summary, our study points to the possibility that the activation of the tumor suppressor LACTB pathway is one of the options for inhibiting EMT program in ovarian cancers via the inhibition of the Slug expression. These notions are supported by previous studies, which showed interconnection of LACTB and EMT also in colorectal and lung cancers (18, 44, 45). In colorectal cancer, it was shown that LACTB inhibits EMT and proliferation through PI3K inactivation, and the use of PI3K inhibitors was suggested as a possible therapeutic intervention (17, 45).

LACTB was previously shown to have connections to lipid metabolism (30). It was identified as a gene associated with lipid metabolism and obesity (31, 32) and its over-expression in transgenic mice led to development of obesity (29). This was followed by our study showing that LACTB’s ability to promote loss of tumorigenicity and onset of differentiation in breast cancer cells is partly dependent on its ability to reprogram the lipid metabolism. This is achieved through LACTB-dependent down-regulation of the lipid-synthesizing mitochondrial PISD enzyme (18). The connection of LACTB and lipid metabolism was further supported by studies in hepatocellular carcinoma where it was shown that LACTB expression influences the expression and activity of important enzymes involved in lipid metabolism (such as carnitine palmitoyl-transferase 1A, acyl-coenzyme A dehydrogenase) (28). Furthermore, recent study by Bennett et al (2022) provides evidence that LACTB filaments can bind liposomes that closely mimic the lipid composition of mitochondrial inner membranes (28). We hypothesize that the interaction of LACTB polymers with inner mitochondrial membranes might modulate the presence and/or activity of proteins/enzymes embedded in this membrane (such as PISD). However, our RNA-seq–based study in ovarian cancer background did not reveal any major changes in pathways involved in lipid metabolism upon LACTB induction, and we did not observe down-regulation of PISD protein upon LACTB induction. These results showed that in contrast to other tissues, LACTB’s differentiation-inducing potential is, in the context of ovary cancer models, manifested through induction of EMT instead of influencing the PISD enzyme and lipid metabolism. Another possible explanation is that even if there are changes in lipid metabolism in ovarian cancer cells upon LACTB induction, these might be mostly manifested at post-transcriptional levels and therefore not possible to detect by RNA-seq analysis. Indeed, metabolic enzymes are known to be often regulated by post-transcriptional and post-translational means to quickly adapt to the metabolic needs of the cells. This aspect will need to be examined more in the future studies.

Our results showed that in contrast to cancer cells, LACTB expression does not lead to down-regulation of the Slug protein in non-tumorigenic cells. These data were also supported by our RNA-seq results, which showed that induction of LACTB in non-tumorigenic immortalized cells does not lead to down-regulation of Slug mRNA. These results resemble the data we observed in breast cancer background, where the differentiation-inducing effects of LACTB were manifested in tumorigenic background and not in the non-tumorigenic controls (18). The reason for the difference in the mechanism of LACTB in the context of non-tumorigenic cell and tumorigenic cells is currently unknown. However, we can speculate that LACTB does not exert these effects in non-tumorigenic cells because non-tumorigenic epithelial cells (of the ovary and breast, where we performed our studies) are already in a differentiated state. As such, the activation of LACTB does not lead to any further differentiation in this context. It is even possible that differentiated tissues express factors that might act as negative modulators of LACTB’s differentiation-inducing ability because this activity is not needed in these tissues anymore. Because tumorigenic cells are often in less differentiated states, LACTB can proceed in these tissues with its differentiation-inducing (whether via lipids or EMT) and tumor suppressive abilities.

In our study, we uncovered the importance of Slug in LACTB’s tumor suppressive mechanism in ovarian cancers. However, more detailed mechanism of the LACTB–Slug axis is currently unknown. Slug is a well-known inhibitor of E-cad (25, 46), what is in agreement with our study showing that LACTB expression leads to decreases in the levels of Slug and increases in the levels of E-cad, an important factor involved in more epithelial, differentiated state of ovary cells. Furthermore, our RNA-seq analysis showed that upon LACTB, induction members of TGFβ and Wnt family are down-regulated, which were previously shown to be positive regulators of Slug expression (47). Therefore, we can hypothesize that LACTB induction leads to inhibition of TGFβ and Wnt pathways, what leads to inhibition of Slug expression and the concomitant increases in E-cad expression. This might ultimately lead to differentiation of ovary cancer cells and their decreased tumorigenicity. However, the precise and detailed mechanism of these and other factors in LACTB biology is currently unknown and will be the focus of future studies. These studies will deepen our understanding of the involvement of the LACTB tumor suppressor pathway in ovarian cancers and might suggest new therapeutic options.

## Materials and Methods

### Cell cultures and reagents

All cells were cultured in 5% CO_2_ humidified incubator at 37°C. The tumorigenic cell lines were purchased, and human primary ovarian epithelial cells were purchased by Cell Biologics and HOSEpC were purchased from iXcells Biotechnologies. FTSEC cell lines (FTSEC190, FTSEC194, FTSEC237), immortalized human fallopian tube epithelial cells (48) were a kind gift from Dr. Ronny I Drapkin (University of Pennsylvania). To activate the tetracycline-inducible gene expression, the cells were treated with 1 μg/ml DOX hyclate (Sigma-Aldrich) in medium. Fresh DOX was added every 3 d. To avoid non-specific DOX effects, our control cells were also treated with DOX where applicable. PEO4, EFO27, KURAMOCHI, OVSAHO, OVCAR8, OVCAR4, OVCAR3, primary, and HOSEpC were cultivated with RPMI 10% FBS and 1% P/S. FTSEC190, FTSEC194, FTSEC237 were cultivated in DMEM F12, 1× UTROSER G (PALL) and 1% P/S (Table S1). Human primary ovarian cells were freshly immortalized in our laboratory through the addition of hTERT-hygro (plasmid #1773; Addgene) to human primary ovarian epithelial cells (cat. H-6036; Cell Biologics Inc.) and selection with hygromycin. Cell lines used in this study were authenticated using STR profiling (Supplemental Data 1).

Table S1 Cell lines.

### Quantitative RT–PCR

Total RNA was isolated directly from cultured cells using the RNeasy Plus Mini kit (74136; Qiagen). Reverse transcription was performed with a high-capacity cDNA Reverse Transcription Kit (4368814; Life Technologies). mRNA levels were measured with gene-specific primers using the SYBR Green I master mix (Roche) on a Roche LightCycler 480 system (Roche). The PCR primer sequences are listed in Table S2. The following genes were evaluated: SLUG, CD44, TGM2, WNT5A, INHBA, LAMA3, GLIPR1, and SERPINE2. After testing with an endogenous gene panel, GAPDH, HPRT ACTIN, HPRT was chosen as reference gene for the normalization.

Table S2 Primers.

The amplification reactions were conducted on a Roche LightCycler 480 system (Roche) using a 384-well plate; each well consisted of 25 ng/ml of cDNA. mRNA levels were measured with gene-specific primers using the SYBR Green I master mix (Roche). The amplification program consisted of an initial cycle of 95°C for 15 s, denaturation at 95°C, followed by 45 cycles of 60°C. After amplification runs, quantification cycle (Cq) values were provided by Roche LightCycler 480 system. Cq value of each sample was normalized using the geometric mean Cq value of the reference gene, corresponding to DCT. Then, the DDCT was calculated, which refers to the Cq of the sample of interest. Finally, the value of 2-DDCt was considered. The obtained data were then further normalized to the expression of these genes relative to the control cells.

### Clinical data in human ovary tissues

LACTB expression was studied on 331 patient samples assembled on a tissue microarray (Biomax). They were all purchased commercially from Biomax (https://www.biomax.us/) as part of their tissue microarray platform. The tissue slide codes are T114a, T113b, OV20810a, OV809b. Tumor tissue was immunohistochemically stained using the LACTB polyclonal antibody (Cat.num. 18195-1-AP, dilution 1:200; Proteintech Group). LACTB expression was analyzed as no expression or lower than in normal tissues (scores 0, 1) and normal expression in the luminal or basal compartment (scores 2, 3).

### In vivo mouse experiments

All manipulations with cells, mice were done in Class II Biological Safety Cabinets to achieve aseptic working environment during the whole study. Cells—PEO4 and PEO4 + LACTB and EFO27 + EFO27 LACTB—were cultivated in RPMI + 10% FBS + 1% penicillin-streptomycin and suspended in 50% matrigel before the application. Adult female mice of strain NSG (NOD.Cg-Prkdcscid Il2rgtm1Wjl/SzJ) were obtained from The Jackson Laboratory. The mice were kept in IVC cages, bedding was changed once weekly, and the mice had access to food and water ad libitum. The mice entered the study when they reached 22 wk. The mice received analgesia before the operation—Rimadyl 2.5 mg/kg and Bupaq 1 mg/kg. For orthotopic tumor application, each mouse was placed into the anesthetic chamber and anesthetized with a mixture of isoflurane and oxygen up to the effect of loss of profound sensitivity. Then, the mice were placed on a heating pad with anesthetization mask. Mice were shaved on the back, and the aseptic operation field was prepared by scrubbing the skin with a cotton swab soaked with povidone–iodine disinfection followed by quaternary ammonium salts disinfection. The operation field was covered by surgical drape. All used surgical equipment was autoclaved before the operation. The injected amount of cells was 2.5 × 10^6^ for EFO27 and EFO27 LACTB and 3 × 10^6^ for PEO4 and PEO4 LACTB prepared in 15 *µ*l for each dose applied with insulin syringe into the ovary fat pad bilaterally via a small surgical wound size of 2 mm close to anatomical position of ovaries. The wound was closed with individual knot stitches in two layers (muscles and subcutaneous tissue) with rapid absorbable surgical material (Chirlac Rapid) size 3/0. The skin was closed with clips, which were removed after 5 d together with remaining suture material.

To monitor mice well-being, we used score sheets which were filled on a daily basis. Body condition score (scale from 1 to 5) and tumor size were measured twice weekly. The in vivo DOX treatment was administered through drinking water containing 2 mg of DOX, 5 mg of glucose, and ½ of tablet of steviol (19 mg of erythritol, 10.5 mg of steviol) in 100 ml of water when most of the tumors reached 4–5 mm in diameter as measured by caliper. The drinking water with doxycycline was changed twice weekly. We measured the tumors on anesthetized mice (with a mixture of air and isoflurane) when the abdominal wall was fully relaxed. The ovaries and the surrounding fat pad, which is bigger than the ovary, were manually palpated on both sides from the backside of mouse. As the tumor grows, the resistance of fat pad tissue and its size become stiffer, and the tumor is better palpable. When the tumor size is between 0.1–0.3 cm, it is measured manually between the thumb and index finger by an experienced person. From ∼0.3 cm tumor size, we measured the tumor size by caliper, where the tumor is gently pulled between jaws of caliper. To obtain the final measurements, it is necessary to deduct 0.3 mm because of the thickness of the abdominal wall. Last tumor measurement was performed on tumors ex vivo. The mice were euthanized when the tumors reached cumulative size of 1.5 cm or earlier when the mice reached humane endpoint. Humane endpoint was set up as a change in body weight more than 20% or BCS 2 or changes in locomotion (scale 1–3) or other health condition change. Sample sizes were chosen to reach statistical significance, and tumor measurements and data analysis were performed in a blinded fashion. At the endpoint of the experiment, tumors were weighted (ex vivo) on an analytical balance (AG204). The animal experiment was approved by the Animal Research Ethics Committee of the Czech Academy of Science, approval ID: AVCR 5282/2021 SOV II.

### Sphere assay

5,000 cells were seeded in ultra-low attachment multiwells (Corning). Spheres were grown in DMEM F12 1% P/S, supplemented with B27 (Gibco), 20 ng/ml EGF, and 10 *µ*g/ml insulin for 10 d (49). Spheres over 50 *µ*M in diameter were counted.

### Immunoblots

Proteins were extracted from cells using RIPA buffer (R0278; Sigma-Aldrich), always in the presence of protease (11836153001; Roche) and phosphatase (04906845001; Roche) inhibitors. Western blots and transfers were performed using standard protocols. Horseradish peroxidase–conjugated secondary antibodies were purchased from Cell Signaling and used at 1:5,000 dilution. Blots were developed using ECL (Dura or Femto; Thermo Fisher Scientific) using the Azure Biosystem c600 equipment.

### Antibodies (Table S3)

The identity, source, catalogue number, and dilution of the antibodies used in this study are summarized in Table S3.

Table S3 Antibodies.

### Flow cytometry (EdU, stem markers)

Cells were trypsinized and filtered through 40-μm cell strainers to obtain single cells. Cells were then labelled for flow cytometry by 1 h incubation with dye-conjugated antibodies and washed once in PBS.

Click-iT EdU Alexa Fluor 647 Flow Cytometry Assay Kit (C10424; Life Technologies) was used for EdU staining according to the manufacturer’s protocol.

### Viral vectors and infections

Human LACTB cDNA was purchased from Open Biosystems (MHS1010-98051227) and subcloned into FUW-tetON lentiviral vector. For lentiviral infection, cells were seeded at 30% confluency in a 10-cm dish and transduced 24 h later with lentiviral vectors in the presence of 5 μg/ml polybrene (TR-1003-G; EMD Millipore). Cells were then selected by the relevant selection marker. Human SLUG cDNA was obtained from Open Biosystems and was subcloned into FUW-LPT2 lentiviral vector (modified from FUW-tetO with puromycin resistance gene). Human shSLUG1 and 2 cloned in the pLKO.1-puro lentiviral vectors were obtained from Addgene (cat #10903 and cat # 10904, respectively).

### Migration assay

Cells were seeded in six-multiwell plates and treated with DOX (1 *µ*g/ml) for 3 d waiting for 100% confluence. Cells were treated with mitomycin (Sigma-Aldrich) (10 *µ*g/ml) for 2 h and after we scratched the surface with a 200-*µ*l tip and took images every 24 h. The area was calculated with ImageJ.

### Immunofluorescence (tissues, cells) and immunohistochemistry

Tumors were fixed in 10% neutral buffered formalin overnight and embedded in paraffin for sectioning. Sections were cut at 5 μm. Tumor sections were deparaffinized in xylene and hydrated in washes with 100%, 96%, 70% ethanol for 1 min. Tissues were boiled in citrate buffer (Sigma-Aldrich) (1×) for 20 min. Slides were treated with 3% H_2_O_2_ at 40°C for 2 min and then washed four times in PBS for 1 min. Blocking solution was added for 20 min (30% horse serum in PBS, 0.1% TWEEN at RT).

Sections were incubated with primary antibody at 4°C overnight. The type, source, and dilution of antibodies are described in Table S3. After three washes with PBS, sections were incubated with secondary antibodies (for IF, AlexaFluor, 1:500; Invitrogen) (for IHC, VECTASTAIN ABC-AP) for 1 h at room temperature, washed three times with PBS, and mounted with Prolong Gold antifade reagent with DAPI (P36931; Life Technologies) and left to dry overnight at room temperature in the dark.

For IHC before mounting, tissues were incubated with solution one-step NBT/BCIP. Slides were dehydrated with 70%, 96%, 100% ethanol and mounted.

Cultured cells were seeded on sterilized, round glass slides inside 6-MW dish dishes with cell culture medium. Cells were washed in PBS, fixed in FIX Buffer1 (BD Biosciences) for 10 min at 37°C, washed three times in PBS, permeabilized with PBS + 0.1% Triton-X for 20 min, washed 3 × 10 min in PBS, and incubated in 5% BSA PBS for 1 h. Cells were then incubated in specific primary antibodies at 4°C overnight, washed three times with PBS, then incubated with secondary antibodies for 1 h at room temperature. After three washes with PBS and one wash with water, slides were mounted on glass microslides with Prolong Gold antifade reagent with DAPI (P36931; Life Technologies) and left to dry overnight at room temperature in the dark. All the tissues used for Fig 1 were bought from BIOMAX (SLIDE CODES: OV809b, OV20810a, T114a, T113b).

### RNA-seq

Total RNA was isolated with GenElute Total RNA Purification Kit (RNB100-100RXN) and subjected to RNA-sequencing core facility at Biocev, Institute of Molecular Genetics, Czech Republic. For the result analysis, the read quality was assessed using FastQC 0.11.8 (https://www.bioinformatics.babraham.ac.uk/projects/fastqc/). Adapters and low-quality sequences were removed using Trimmomatic 0.39 (50). Clean reads were then mapped into the hg38 human genome using HISAT 2.1.0 (51) and Samtools 1.10 (52). Read coverage profiles were then computed using the bamCoverage tool from the deepTools 3.5.1 package (53). Mapped reads and coverage data were inspected visually in the IGV 2.9 browser (54). Differentially expressed genes were detected using the DESeq2 package (55) in R/Bioconductor (56, 57). All scripts and code are available from GitHub at https://github.com/mprevorovsky/keckesova-LACTB.

Heat map was generated from the output of DESeq2 analysis performed on the RNA-seq data, with genes considered statistically significant (*P* < 0.05). Z-scores were computed from the normalized gene counts.

(ArrayExpress accession number E-MTAB-12053).

### Statistics

Results were expressed as means ± SDs for at least three independent experiments. Statistical analysis was performed using two-way ANOVA and *t* test with the level of significance set at *P* < 0.05. *P < 0.05; **P < 0.01; ***P < 0.005, ****P < 0.001.

Supplemental Data 1. STR profile of the cells.

## Data Availability

The accession number for the RNA-sequencing data reported in this paper is E-MTAB-12053 (Array Express).

## Supplementary Material

Reviewer comments
