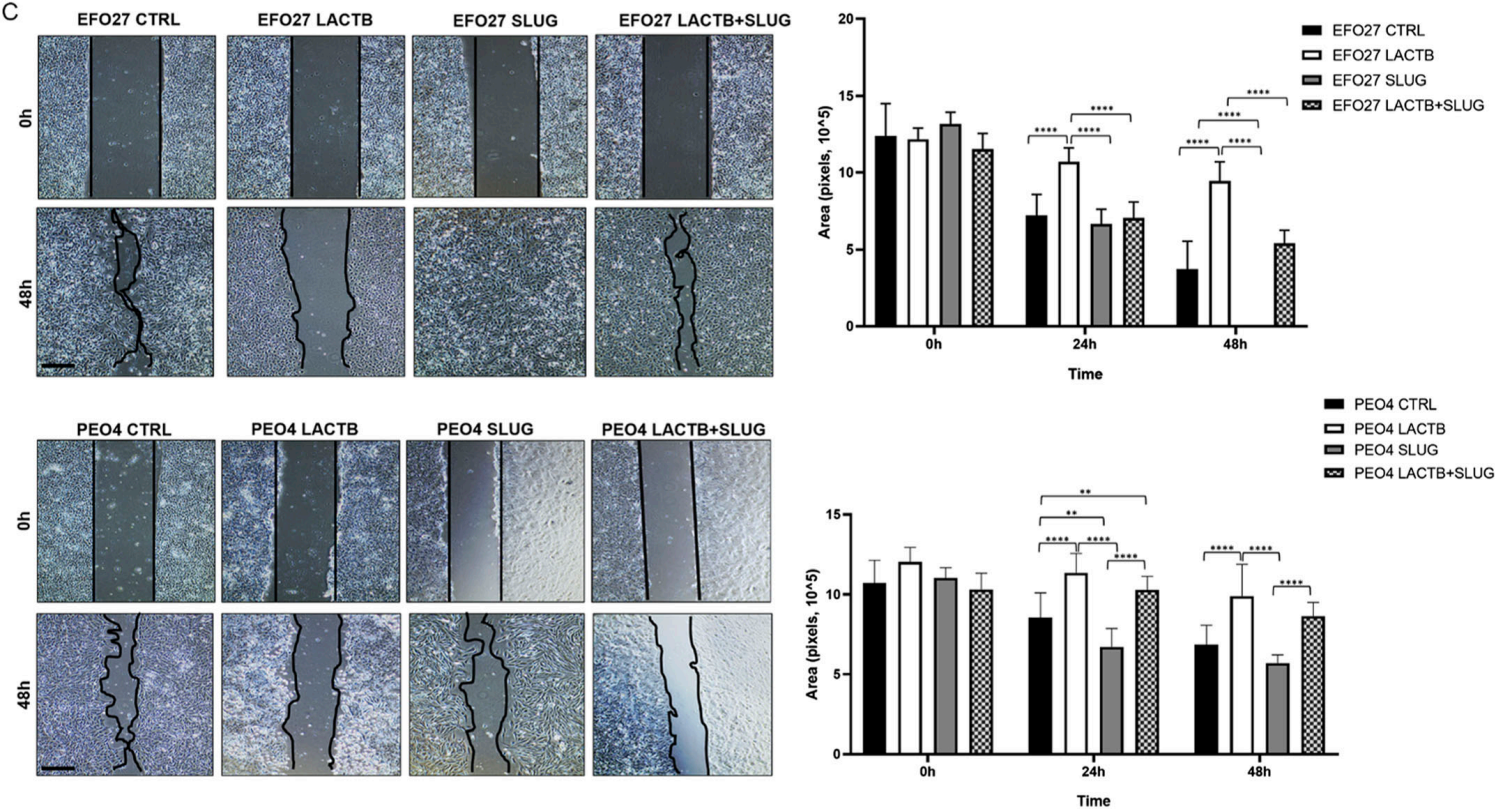# Correction: LACTB exerts tumor suppressor properties in epithelial ovarian cancer through regulation of Slug

**DOI:** 10.26508/lsa.202302497

**Published:** 2023-12-06

**Authors:** Valentina Cutano, Jessica Marianne Ferreira Mendes, Sara Escudeiro-Lopes, Susana Machado, Judith Vinaixa Forner, Juan M Gonzales-Morena, Martin Prevorovsky, Viacheslav Zemlianski, Yuxiong Feng, Petra Kralova Viziova, Andrea Hartmanova, Beata Malcekova, Pavel Jakoube, Sonia Iyer, Zuzana Keckesova

**Affiliations:** 1 Institute of Organic Chemistry and Biochemistry, Czech Academy of Sciences , Prague, Czech Republic; 2 Department of Cell Biology, Faculty of Science, Charles University, Prague, Czech Republic; 3 Zhejiang Provincial Key Laboratory of Pancreatic Disease, First Affiliated Hospital, and Institute of Translational Medicine, Zhejiang University School of Medicine, Hangzhou, China; 4 The Czech Center for Phenogenomics, Institute of Molecular Genetics of the Czech Academy of Sciences, Vestec, Czech Republic; 5 Whitehead Institute for Biomedical Research, Cambridge, MA, USA

## Abstract

We show the ability of LACTB to function as a tumor suppressor in ovarian cancer through down-regulation of Slug and induction of differentiation.

Article: Cutano V, Ferreira Mendes JM, Escudeiro-Lopes S, Machado S, Vinaixa Forner J, Gonzales-Morena JM, Prevorovsky M, Zemlianski V, Feng Y, Kralova Viziova P, Hartmanova A, Malcekova B, Jakoube P, Iyer S, Keckesova Z (2022 Nov 14) LACTB exerts tumor suppressor properties in epithelial ovarian cancer through regulation of Slug. Life Science Alliance 6(1): e202201510. doi: 10.26508/lsa.202201510. PMID: 36375842.

Correction Fig 6C: In our article, there is an accidental duplication of two neighboring representative images in Fig 6C, where image labelled “PEO4 LACTB + SLUG, 0 h” is a duplication of neighboring image “PEO4 SLUG, 0 h.” In this correction, we have replaced the faulty image “PEO4 LACTB + SLUG, 0 h” with the correct one. Associated quantifications and figure conclusions were not affected by this image duplication.

**Figure 6.**