# Supplementary material for: LACTB exerts tumor suppressor properties in epithelial ovarian cancer through regulation of Slug
Source: Life Sci Alliance. 2022 Nov 14;6(1):e202201510. doi: 10.26508/lsa.202201510 (PMC9664245; doi:10.26508/lsa.202201510)
Supplement: Supplementary file 2 [file LSA-2022-01510_TableS1.docx]

**Supplementary Table 1 (Cell lines)**

| **CELL LINE** | **RRID CODE** | **DISEASE** | **ORIGIN** | **CATALOG NUMBER** |
| --- | --- | --- | --- | --- |
| KURAMOCHI | CVCL_1345 | High grade ovarian serous adenocarcinoma | JCRB | 0098 |
| OVSAHO | CVCL_3114 | High grade ovarian serous adenocarcinoma | Sigma Aldrich | SCC294 |
| EFO27 | CVCL_1192 | Ovarian mucinous adenocarcinoma | Creative bioarray | CSC-C0318 |
| PEO4 | CVCL_2690 | Ovarian cystadenocarcinoma | Sigma Aldrich | 10032309-1VL |
| OVCAR8 | CVCL_1629 | High grade ovarian serous adenocarcinoma | NCI-60 CANCER PANEL |  |
| JHOS4 | CVCL_4649 | High grade ovarian serous adenocarcinoma | Creative bioarray | CSC-C6613J |
| OVCAR4 | CVCL_1627 | High grade ovarian serous adenocarcinoma | NCI-60 CANCER PANEL |  |
| OVCAR3 | CVCL_0465 | High grade ovarian serous adenocarcinoma | NCI-60 CANCER PANEL |  |
| HOSEpC | / | Ovary | iXcells biotech | 10HU-107 |
| Human Primary Ovarian Epithelial Cells | / | Ovary | Cell biologics | H-6036 |
| Imm. Primary cells | / | Ovary | Keckesova's Lab |  |
| FTSEC 190 | / | Fallopian tubes | Drapkin's Lab |  |
| FTSEC 194 | / | Fallopian tubes | Drapkin's Lab |  |
| FTSEC 237 | / | Fallopian tubes | Drapkin's Lab |  |
