# Supplementary material for: LACTB exerts tumor suppressor properties in epithelial ovarian cancer through regulation of Slug
Source: Life Sci Alliance. 2022 Nov 14;6(1):e202201510. doi: 10.26508/lsa.202201510 (PMC9664245; doi:10.26508/lsa.202201510)
Supplement: Supplementary file 3 [file LSA-2022-01510_TableS2.docx]

**Supplementary Table 2 (PRIMERS)**

| **GENE** | **FW** | **RV** |
| --- | --- | --- |
| LACTB | GTGGTTGGAGTTTCTGTAGATGGAA | AGTAATCTTGTTGTGACAGAAACCT |
| SLUG | GTTTCAGTGCAATTTATGCA | TTCTAATGTGTCCTTGAAGC |
| GLIPR1 | GGCTGCGCAGTTCAATTTTG | GTCATTATTGGGGCAGGCAC |
| CD44 | CGCCAAACACCCAAAGAAGA | TTCCTGCTTGATGACCTCGT |
| TGM2 | AGGCCCGTTTTCCACTAAGA | AGCAAAATGAAGTGGCCCAG |
| SERPINE2 | TTGTGAAGTCGAGGCCTCAT | TTCTTGGAGACGATGGCCTT |
| WNT7A | TTCACCTACGCCATCATTGC | TTCATGAGAGTCCGGGCATT |
| INHBA | TGTACCCAACTCTCAGCCAG | TGCCCTCCTTCCAATGTCAT |
| LAMA3 | CAGGGCCAGTTCTGTGACTA | CCCAGATCCAAGGTGAGGTT |
| HPRT | AGGCCATCACATTGTAGCCC | GTCCCCTGTTGACTGGTCATT |
