# Supplementary material for: LACTB exerts tumor suppressor properties in epithelial ovarian cancer through regulation of Slug
Source: Life Sci Alliance. 2022 Nov 14;6(1):e202201510. doi: 10.26508/lsa.202201510 (PMC9664245; doi:10.26508/lsa.202201510)
Supplement: Supplementary file 4 [file LSA-2022-01510_TableS3.docx]

**Supplementary Table 3 (ANTIBODIES)**

| **Table 3** | **DILUTION:** | |  |  |  |
| --- | --- | --- | --- | --- | --- |
| **Antibody_Cat. Number_Brand** | **WB** | **IF** | **IHC** | **FACS** |  |
| LACTB (18195-1-AP) ProteinTech | 1:1000 | 1:250 | 1:200 |  |  |
| GAPDH (14C10) Cell Signaling | 1:1000 |  |  |  |  |
| Ki67 (550609) BD Pharmingen |  | 1:100 |  |  |  |
| CD44-PE (338808) Biolegend |  |  |  | 1:400 |  |
| ALDH1A1 (15910-1-AP) ProteinTech |  |  |  | 1:800 |  |
| E-CAD (20874-1-AP) ProteinTech |  | 1:200 |  |  |  |
| SLUG (C19G7) Cell Signaling | 1:1000 |  |  |  |  |
| PISD (16401-1-AP) ProteinTech  40KDa band | 1:1000 |  |  |  |  |
