# Supplementary material for: LACTB exerts tumor suppressor properties in epithelial ovarian cancer through regulation of Slug
Source: Life Sci Alliance. 2022 Nov 14;6(1):e202201510. doi: 10.26508/lsa.202201510 (PMC9664245; doi:10.26508/lsa.202201510)
Supplement: Supplementary file 5 [file LSA-2022-01510_Supplemental_Data_1.pdf]

GENERI BIOTECH s.r.o.
Testovací laboratoř
Machkova 587/42
500 11 Hradec Králové - Třebeš

# Report no. 5359-2/2022

| | |
|---|---|
| **Test:** | Cell line authentication |
| **Testing place:** | Testovací laboratoř |
| **Case marking:** | 5359-2/2022 |
| **Sample marking:** | FTSEC194+TetON Adv |
| **Contracting authority*:** | Zuzana Kečkéšová |
| **Address*:** | ÚOCHB<br>Flemingovo Náměstí 2<br>Praha<br>166 10 |
| **Sample type:** | DNA isolate |
| **Sample coll. method:** | by client |
| **Number of samples:** | 2 |

| Sample marking* | sampling date/passage* | receive date | received by |
|---|---|---|---|
| **FTSEC194+TetON Adv** | 03.10.2022/ N/A | 11.10.2022 | Šmí |

**Analysis date:** 11.10.2022 - 14.10.2022

**Used methods:** SOP_T1_014 Autentizace_buněčných_linií (STR profil)_fragmentační_analýza_DNA

**Test result:**
Cell line marked FTSEC194+TetON Adv is identical to reference cell line hTERT FT 194 (CRL-3445)
No. of mismatched alleles 0. Conformity level is 100 %.
According to ICLAC guide, cell line is authenticated when conformity level is more than 80 %.

The test results are applicable to tested samples only (in the condition as delivered).

* information from the contracting authority, Testing laboratory is not responsible for this information

**Report date:** 14.10.2022

**Person responsible for the test**

Mgr. Dagmar Hodíková
Deputy head of Testing laboratory

Mgr. Dagmar Hodíková
Digitálně podepsal Mgr. Dagmar Hodíková
Datum: 2022.10.14 15:35:08 +02'00'

**Report released by**

Mgr. Veronika Šmídová
Head of Testing laboratory

Mgr. Veronika Šmídová
Digitálně podepsal Mgr. Veronika Šmídová
Datum: 2022.10.17 13:13:20 +02'00'

Report no. 5359-2/2022

GENERI BIOTECH s.r.o.
Testovací laboratoř
Machkova 587/42
500 11 Hradec Králové - Třebeš

| Polymorfism | Sample* | | | Reference cell line | | | Degree of compliance | |
|---|---|---|---|---|---|---|---|---|
| | FTSEC194+TetON Adv | | | hTERT FT 194 CRL-3445 | | | | |
| Amelogenin | X | X | | X | X | | + | + |
| CSF1PO | 11 | 13 | | 11 | 13 | | + | + |
| D13S317 | 11 | 11 | | 11 | 11 | | + | + |
| D16S539 | 11 | 12 | | 11 | 12 | | + | + |
| D18S51 | 13 | 18 | | 0 | 0 | | N/A | N/A |
| D19S433 | 13 | 15 | | 0 | 0 | | N/A | N/A |
| D21S11 | 30 | 30 | | 0 | 0 | | N/A | N/A |
| D2S1338 | 17 | 20 | | 0 | 0 | | N/A | N/A |
| D3S1358 | 14 | 18 | | 0 | 0 | | N/A | N/A |
| D5S818 | 11 | 12 | | 11 | 12 | | + | + |
| D7S820 | 10 | 13 | | 10 | 13 | | + | + |
| D8S1179 | 13 | 15 | | 0 | 0 | | N/A | N/A |
| FGA | 24 | 25 | | 0 | 0 | | N/A | N/A |
| Penta D | 0 | 0 | | 0 | 0 | | N/A | N/A |
| Penta E | 0 | 0 | | 0 | 0 | | N/A | N/A |
| TH01 | 8 | 9.3 | | 8 | 9.3 | | + | + |
| TPOX | 8 | 8 | | 8 | 8 | | + | + |
| vWA | 16 | 17 | | 16 | 17 | | + | + |

No. of mismatches: 0
Conformity level: 100%

Used database: ATCC

* information from the contracting authority, Testing laboratory is not responsible for this information

Report no. 5359-2/2022

Testing laboratory no. 1650 accredited ČIA according to ČSN EN ISO/IEC 17025:2018

GENERI BIOTECH s.r.o.
Testovací laboratoř
Machkova 587/42
500 11 Hradec Králové - Třebeš

L 1650

# Report no. 5225-5/2022

| | |
|---|---|
| **Test:** | Cell line authentication |
| **Testing place:** | Testovací laboratoř |
| **Case marking:** | 5225-5/2022 |
| **Sample marking:** | OVSAHO |
| **Contracting authority*:** | Zuzana Kečkéšová |
| **Address*:** | ÚOCHB<br>Flemingovo Náměstí 2<br>Praha<br>166 10 |
| **Sample type:** | DNA isolate |
| **Sample coll. method:** | by client |
| **Number of samples:** | 11 |

| Sample marking* | sampling date/passage* | receive date | received by |
|---|---|---|---|
| **OVSAHO** | N/A | 24.08.2022 | Hod |

**Analysis date:** 24.08.2022 - 02.09.2022

**Used methods:** SOP_T1_014 Autentizace_buněčných_linií (STR profil)_fragmentační_analýza_DNA

**Test result:**
Cell line marked OVSAHO is identical to reference cell line OVSAHO (CVCL_3114)
No. of mismatched alleles 0. Conformity level is 100 %.
According to ICLAC guide, cell line is authenticated when conformity level is more than 80 %.

The test results are applicable to tested samples only (in the condition as delivered).

* information from the contracting authority, Testing laboratory is not responsible for this information

**Report date:** 02.09.2022

**Person responsible for the test**

Mgr. Dagmar Hodíková
Deputy head of Testing laboratory

Mgr. Dagmar Hodíková
Digitálně podepsal Mgr. Dagmar Hodíková
Datum: 2022.09.02 13:11:10 +02'00'

**Report released by**

Mgr. Veronika Šmídová
Head of Testing laboratory

Mgr. Veronika Šmídová
Digitálně podepsal Mgr. Veronika Šmídová
Datum: 2022.09.05 10:27:07 +02'00'

| Polymorfism | Sample* | | | Reference cell line | | | Degree of compliance | |
|---|---|---|---|---|---|---|---|---|
| | OVSAHO | | | OVSAHO CVCL_3114 | | | | |
| Amelogenin | X | X | | X | X | | + | + |
| CSF1PO | 10 | 12 | | 10 | 12 | | + | + |
| D13S317 | 8 | 8 | | 8 | 8 | | + | + |
| D16S539 | 9 | 9 | | 9 | 9 | | + | + |
| D18S51 | 16 | 20 | | 16 | 20 | | + | + |
| D19S433 | 15.2 | 16.2 | | 0 | 0 | | N/A | N/A |
| D21S11 | 31 | 31 | | 31 | 31 | | + | + |
| D2S1338 | 23 | 23 | | 23 | 23 | | + | + |
| D3S1358 | 15 | 15 | | 15 | 15 | | + | + |
| D5S818 | 12 | 13 | | 12 | 13 | | + | + |
| D7S820 | 8 | 10 | | 8 | 10 | | + | + |
| D8S1179 | 14 | 14 | | 14 | 14 | | + | + |
| FGA | 24 | 24 | | 24 | 24 | | + | + |
| Penta D | 0 | 0 | | 9 | 9 | | N/A | N/A |
| Penta E | 0 | 0 | | 15 | 15 | | N/A | N/A |
| TH01 | 6 | 6 | | 6 | 6 | | + | + |
| TPOX | 8 | 11 | | 8 | 11 | | + | + |
| vWA | 14 | 14 | | 14 | 14 | | + | + |

No. of mismatches: 0
Conformity level: 100%

Used database: Expasy - Cellosaurus

* information from the contracting authority, Testing laboratory is not responsible for this information

Report no. 5225-5/2022

GENERI BIOTECH s.r.o.
Testovací laboratoř
Machkova 587/42
500 11 Hradec Králové - Třebeš

L 1650

# Report no. 5225-3/2022

**Test:** Cell line authentication
**Testing place:** Testovací laboratoř
**Case marking:** 5225-3/2022
**Sample marking:** Kuramochi
**Contracting authority*:** Zuzana Kečkéšová
**Address*:** ÚOCHB
Flemingovo Náměstí 2
Praha
166 10
**Sample type:** DNA isolate
**Sample coll. method:** by client
**Number of samples:** 11

| Sample marking* | sampling date/passage* | receive date | received by |
|---|---|---|---|
| **Kuramochi** | N/A | 24.08.2022 | Hod |

**Analysis date:** 24.08.2022 - 05.09.2022

**Used methods:** SOP_T1_014 Autentizace_buněčných_linií (STR profil)_fragmentační_analýza_DNA

**Test result:**
Cell line marked Kuramochi is identical to reference cell line Kuramochi (CVCL_1345)
No. of mismatched alleles 0. Conformity level is 100 %.
According to ICLAC guide, cell line is authenticated when conformity level is more than 80 %.

The test results are applicable to tested samples only (in the condition as delivered).

* information from the contracting authority, Testing laboratory is not responsible for this information

**Report date:** 05.09.2022

**Person responsible for the test**

Mgr. Dagmar Hodíková
Deputy head of Testing laboratory

Mgr. Dagmar Hodíková
Digitálně podepsal Mgr. Dagmar Hodíková
Datum: 2022.09.05 10:26:56 +02'00'

**Report released by**

Mgr. Veronika Šmídová
Head of Testing laboratory

Mgr. Veronika Šmídová
Digitálně podepsal Mgr. Veronika Šmídová
Datum: 2022.09.05 10:28:52 +02'00'

| Polymorfism | Sample* | | | Reference cell line | | | Degree of compliance | |
|---|---|---|---|---|---|---|---|---|
| | Kuramochi | | | Kuramochi CVCL_1345 | | | | |
| Amelogenin | X | X | | X | X | | + | + |
| CSF1PO | 11 | 12 | | 11 | 12 | | + | + |
| D13S317 | 9 | 12 | | 9 | 12 | | + | + |
| D16S539 | 10 | 10 | | 10 | 10 | | + | + |
| D18S51 | 13 | 13 | | 13 | 13 | | + | + |
| D19S433 | 13 | 14 | | 0 | 0 | | N/A | N/A |
| D21S11 | 30 | 32.2 | | 30 | 32.2 | | + | + |
| D2S1338 | 18 | 18 | | 0 | 0 | | N/A | N/A |
| D3S1358 | 18 | 18 | | 18 | 18 | | + | + |
| D5S818 | 12 | 12 | | 12 | 12 | | + | + |
| D7S820 | 10 | 11 | | 10 | 11 | | + | + |
| D8S1179 | 10 | 11 | | 10 | 11 | | + | + |
| FGA | 21 | 23 | | 21 | 23 | | + | + |
| Penta D | 0 | 0 | | 10 | 13 | | N/A | N/A |
| Penta E | 0 | 0 | | 15 | 15 | | N/A | N/A |
| TH01 | 9 | 9 | | 9 | 9 | | + | + |
| TPOX | 8 | 12 | | 8 | 12 | | + | + |
| vWA | 16 | 19 | | 16 | 19 | | + | + |

No. of mismatches: 0
Conformity level: 100%

Used database: Expasy - Cellosaurus

* information from the contracting authority, Testing laboratory is not responsible for this information

Report no. 5225-3/2022

GENERI BIOTECH s.r.o.
Testovací laboratoř
Machkova 587/42
500 11 Hradec Králové - Třebeš

L 1650

# Report no. 5225-1/2022

**Test:** Cell line authentication
**Testing place:** Testovací laboratoř
**Case marking:** 5225-1/2022
**Sample marking:** EFO27
**Contracting authority*:** Zuzana Kečkéšová
**Address*:** ÚOCHB
Flemingovo Náměstí 2
Praha
166 10
**Sample type:** DNA isolate
**Sample coll. method:** by client
**Number of samples:** 11

| Sample marking* | sampling date/passage* | receive date | received by |
| --- | --- | --- | --- |
| **EFO27** | N/A | 24.08.2022 | Hod |

**Analysis date:** 24.08.2022 - 05.09.2022

**Used methods:** SOP_T1_014 Autentizace_buněčných_linií (STR profil)_fragmentační_analýza_DNA

**Test result:**
Cell line marked EFO27 is identical to reference cell line EFO27 (CVCL_1192)
No. of mismatched alleles 4. Conformity level is 87 %.
According to ICLAC guide, cell line is authenticated when conformity level is more than 80 %.

The test results are applicable to tested samples only (in the condition as delivered).

* information from the contracting authority, Testing laboratory is not responsible for this information

**Report date:** 05.09.2022

**Person responsible for the test**

Mgr. Dagmar Hodíková
Deputy head of Testing laboratory

Mgr. Dagmar Hodíková
Digitálně podepsal Mgr. Dagmar Hodíková
Datum: 2022.09.05 10:20:57 +02'00'

**Report released by**

Mgr. Veronika Šmídová
Head of Testing laboratory

Mgr. Veronika Šmídová
Digitálně podepsal Mgr. Veronika Šmídová
Datum: 2022.09.05 10:22:58 +02'00'

| Polymorfism | Sample* | | | Reference cell line | | | Degree of compliance | |
|---|---|---|---|---|---|---|---|---|
| | EFO27 | | | EFO27<br>CVCL_1192 | | | | |
| Amelogenin | X | X | | X | X | | + | + |
| CSF1PO | 9 | 10 | 13,14,15 | 9 | 10,13 | 14 | + | - |
| D13S317 | 12 | 13 | | 12 | 13 | | + | + |
| D16S539 | 11 | 13 | | 11 | 13 | | + | + |
| D18S51 | 12 | 18 | | 12 | 18 | | + | + |
| D19S433 | 13 | 14 | | 13 | 14 | | + | + |
| D21S11 | 27 | 30 | 31 | 27 | 30 | 31 | + | + |
| D2S1338 | 22 | 25 | | 22 | 25 | | + | + |
| D3S1358 | 14 | 17 | | 14 | 17 | | + | + |
| D5S818 | 11 | 12 | | 11 | 12 | | + | + |
| D7S820 | 8 | 9 | 10 | 8 | 10 | | + | - |
| D8S1179 | 13 | 14 | 15 | 13 | 15 | | + | - |
| FGA | 23 | 25 | 26 | 23 | 26 | | + | - |
| Penta D | 0 | 0 | | 9 | 9 | | N/A | N/A |
| Penta E | 0 | 0 | | 7 | 10 | | N/A | N/A |
| TH01 | 6 | 8 | | 6 | 8 | | + | + |
| TPOX | 8 | 8 | | 8 | 8 | | + | + |
| vWA | 13 | 18 | | 13 | 18 | | + | + |

No. of mismatches: 4
Conformity level: 87%

Used database: Expasy - Cellosaurus

* information from the contracting authority, Testing laboratory is not responsible for this information

Report no. 5225-1/2022

GENERI BIOTECH s.r.o.
Testovací laboratoř
Machkova 587/42
500 11 Hradec Králové - Třebeš

L 1650

# Report no. 5225-7/2022

| | |
|---|---|
| **Test:** | Cell line authentication |
| **Testing place:** | Testovací laboratoř |
| **Case marking:** | 5225-7/2022 |
| **Sample marking:** | PEO4 |
| **Contracting authority*:** | Zuzana Kečkéšová |
| **Address*:** | ÚOCHB<br>Flemingovo Náměstí 2<br>Praha<br>166 10 |
| **Sample type:** | DNA isolate |
| **Sample coll. method:** | by client |
| **Number of samples:** | 11 |

| Sample marking* | sampling date/passage* | receive date | received by |
|---|---|---|---|
| **PEO4** | N/A | 24.08.2022 | Hod |

**Analysis date:** 24.08.2022 - 02.09.2022

**Used methods:** SOP_T1_014 Autentizace_buněčných_linií (STR profil)_fragmentační_analýza_DNA

**Test result:**
Cell line marked PEO4 is identical to reference cell line PEO4 (CVCL_2690)
No. of mismatched alleles 1. Conformity level is 96 %.
According to ICLAC guide, cell line is authenticated when conformity level is more than 80 %.

The test results are applicable to tested samples only (in the condition as delivered).

* information from the contracting authority, Testing laboratory is not responsible for this information

**Report date:** 02.09.2022

**Person responsible for the test**

Mgr. Dagmar Hodíková
Deputy head of Testing laboratory

Mgr. Dagmar Hodíková
Digitálně podepsal Mgr. Dagmar Hodíková
Datum: 2022.09.02 10:31:26 +02'00'

**Report released by**

Mgr. Veronika Šmídová
Head of Testing laboratory

Mgr. Veronika Šmídová
Digitálně podepsal Mgr. Veronika Šmídová
Datum: 2022.09.05 10:47:43 +02'00'

| Polymorfism | Sample* | | | Reference cell line | | | Degree of compliance | |
|---|---|---|---|---|---|---|---|---|
| | PEO4 | | | PEO4 CVCL_2690 | | | | |
| Amelogenin | X | X | | X | X | | + | + |
| CSF1PO | 10 | 12 | | 10 | 12 | | + | + |
| D13S317 | 10 | 10 | | 10 | 10 | | + | + |
| D16S539 | 9 | 9 | | 9 | 9 | | + | + |
| D18S51 | 16 | 17 | | 16 | 17 | | + | + |
| D19S433 | 13 | 15 | | 13 | 15 | | + | + |
| D21S11 | 32.2 | 32.2 | | 30 | 32.2 | | - | + |
| D2S1338 | 20 | 21 | | 20 | 21 | | + | + |
| D3S1358 | 16 | 16 | | 16 | 16 | | + | + |
| D5S818 | 11 | 12 | | 11 | 12 | | + | + |
| D7S820 | 10 | 10 | | 10 | 10 | | + | + |
| D8S1179 | 13 | 14 | | 13 | 14 | | + | + |
| FGA | 20 | 20 | | 20 | 20 | | + | + |
| Penta D | 0 | 0 | | 9 | 14 | | N/A | N/A |
| Penta E | 0 | 0 | | 11 | 12 | | N/A | N/A |
| TH01 | 9.3 | 9.3 | | 9.3 | 9.3 | | + | + |
| TPOX | 9 | 11 | | 9 | 11 | | + | + |
| vWA | 15 | 16 | | 15 | 16 | | + | + |

No. of mismatches: 1
Conformity level: 96%

Used database: Expasy - Cellosaurus

* information from the contracting authority, Testing laboratory is not responsible for this information

Report no. 5225-7/2022

Testing laboratory no. 1650 accredited ČIA according to ČSN EN ISO/IEC 17025:2018

GENERI BIOTECH s.r.o.
Testovací laboratoř
Machkova 587/42
500 11 Hradec Králové - Třebeš

L 1650

# Report no. 5225-4/2022

**Test:** Cell line authentication
**Testing place:** Testovací laboratoř
**Case marking:** 5225-4/2022
**Sample marking:** OVCAR8
**Contracting authority*:** Zuzana Kečkéšová
**Address*:** ÚOCHB
Flemingovo Náměstí 2
Praha
166 10
**Sample type:** DNA isolate
**Sample coll. method:** by client
**Number of samples:** 11

| Sample marking* | sampling date/passage* | receive date | received by |
| --- | --- | --- | --- |
| **OVCAR8** | N/A | 24.08.2022 | Hod |

**Analysis date:** 24.08.2022 - 02.09.2022

**Used methods:** SOP_T1_014 Autentizace_buněčných_linií (STR profil)_fragmentační_analýza_DNA

**Test result:**
Cell line marked OVCAR8 is identical to reference cell line OVCAR8 (CVCL_1629)
No. of mismatched alleles 0. Conformity level is 100 %.
According to ICLAC guide, cell line is authenticated when conformity level is more than 80 %.

The test results are applicable to tested samples only (in the condition as delivered).

* information from the contracting authority, Testing laboratory is not responsible for this information

**Report date:** 02.09.2022

**Person responsible for the test**

Mgr. Dagmar Hodíková
Deputy head of Testing laboratory

Mgr. Dagmar Hodíková
Digitálně podepsal Mgr. Dagmar Hodíková
Datum: 2022.09.02 13:08:11 +02'00'

**Report released by**

Mgr. Veronika Šmídová
Head of Testing laboratory

Mgr. Veronika Šmídová
Digitálně podepsal Mgr. Veronika Šmídová
Datum: 2022.09.05 10:25:02 +02'00'

| Polymorfism | Sample* | | | Reference cell line | | | Degree of compliance | |
|---|---|---|---|---|---|---|---|---|
| | OVCAR8 | | | OVCAR8 CVCL_1629 | | | | |
| Amelogenin | X | X | | X | X | | + | + |
| CSF1PO | 11 | 11 | | 11 | 11 | | + | + |
| D13S317 | 12 | 12 | | 12 | 12 | | + | + |
| D16S539 | 13 | 13 | | 13 | 13 | | + | + |
| D18S51 | 14 | 14 | | 14 | 14 | | + | + |
| D19S433 | 14 | 16 | | 14 | 16 | | + | + |
| D21S11 | 28 | 28 | | 28 | 28 | | + | + |
| D2S1338 | 19 | 23 | | 19 | 23 | | + | + |
| D3S1358 | 16 | 18 | | 16 | 18 | | + | + |
| D5S818 | 12 | 12 | | 12 | 12 | | + | + |
| D7S820 | 12 | 12 | | 12 | 12 | | + | + |
| D8S1179 | 10 | 10 | | 10 | 10 | | + | + |
| FGA | 20 | 20 | | 20 | 20 | | + | + |
| Penta D | 0 | 0 | | 12 | 12 | | N/A | N/A |
| Penta E | 0 | 0 | | 10 | 10 | | N/A | N/A |
| TH01 | 7 | 7 | | 7 | 7 | | + | + |
| TPOX | 8 | 8 | | 8 | 8 | | + | + |
| vWA | 16 | 17 | | 16 | 17 | | + | + |

No. of mismatches: 0
Conformity level: 100%

Used database: Expasy - Cellosaurus

* information from the contracting authority, Testing laboratory is not responsible for this information

Report no. 5225-4/2022

GENERI BIOTECH s.r.o.
Testovací laboratoř
Machkova 587/42
500 11 Hradec Králové - Třebeš

L 1650

# Report no. 5225-10/2022

| | |
|---|---|
| **Test:** | Cell line authentication |
| **Testing place:** | Testovací laboratoř |
| **Case marking:** | 5225-10/2022 |
| **Sample marking:** | FTSEC237 |
| **Contracting authority*:** | Zuzana Kečkéšová |
| **Address*:** | ÚOCHB<br>Flemingovo Náměstí 2<br>Praha<br>166 10 |
| **Sample type:** | DNA isolate |
| **Sample coll. method:** | by client |
| **Number of samples:** | 11 |

| Sample marking* | sampling date/passage* | receive date | received by |
|---|---|---|---|
| **FTSEC237** | N/A | 24.08.2022 | Hod |

**Analysis date:** 24.08.2022 - 02.09.2022

**Used methods:** SOP_T1_014 Autentizace_buněčných_linií (STR profil)_fragmentační_analýza_DNA

**Test result:**
Cell line marked FTSEC237 is identical to reference cell line hTERT FT 237 (CRL-3446)
No. of mismatched alleles 0. Conformity level is 100 %.
According to ICLAC guide, cell line is authenticated when conformity level is more than 80 %.

The test results are applicable to tested samples only (in the condition as delivered).

* information from the contracting authority, Testing laboratory is not responsible for this information

**Report date:** 02.09.2022

**Person responsible for the test**

Mgr. Dagmar Hodíková
Deputy head of Testing laboratory

Mgr. Dagmar Hodíková
Digitálně podepsal Mgr. Dagmar Hodíková
Datum: 2022.09.20 10:43:19 +02'00'

**Report released by**

Mgr. Veronika Šmídová
Head of Testing laboratory

Mgr. Veronika Šmídová
Digitálně podepsal Mgr. Veronika Šmídová
Datum: 2022.09.20 12:17:22 +02'00'

| Polymorfism | Sample* | | | Reference cell line | | | Degree of compliance | |
|---|---|---|---|---|---|---|---|---|
| | FTSEC237 | | | hTERT FT 237 CRL-3446 | | | | |
| Amelogenin | X | X | | X | X | | + | + |
| CSF1PO | 10 | 10 | | 10 | 10 | | + | + |
| D13S317 | 9 | 9 | | 9 | 9 | | + | + |
| D16S539 | 9 | 11 | | 9 | 11 | | + | + |
| D18S51 | 12 | 17 | | 0 | 0 | | N/A | N/A |
| D19S433 | 14 | 15 | | 0 | 0 | | N/A | N/A |
| D21S11 | 32.2 | 33.2 | | 0 | 0 | | N/A | N/A |
| D2S1338 | 24 | 24 | | 0 | 0 | | N/A | N/A |
| D3S1358 | 14 | 18 | | 0 | 0 | | N/A | N/A |
| D5S818 | 12 | 12 | | 12 | 12 | | + | + |
| D7S820 | 7 | 12 | | 7 | 12 | | + | + |
| D8S1179 | 13 | 15 | | 0 | 0 | | N/A | N/A |
| FGA | 22 | 25 | | 0 | 0 | | N/A | N/A |
| Penta D | 0 | 0 | | 0 | 0 | | N/A | N/A |
| Penta E | 0 | 0 | | 0 | 0 | | N/A | N/A |
| TH01 | 6 | 9.3 | | 6 | 9.3 | | + | + |
| TPOX | 9 | 11 | | 9 | 11 | | + | + |
| vWA | 16 | 17 | | 16 | 17 | | + | + |

No. of mismatches: 0
Conformity level: 100%

Used database: ATCC

* information from the contracting authority, Testing laboratory is not responsible for this information

Report no. 5225-10/2022

Testing laboratory no. 1650 accredited ČIA according to ČSN EN ISO/IEC 17025:2018

GENERI BIOTECH s.r.o.
Testovací laboratoř
Machkova 587/42
500 11 Hradec Králové - Třebeš

L 1650

# Report no. 5225-9/2022

| | |
|---|---|
| **Test:** | Cell line authentication |
| **Testing place:** | Testovací laboratoř |
| **Case marking:** | 5225-9/2022 |
| **Sample marking:** | FTSEC190 |
| **Contracting authority*:** | Zuzana Kečkéšová |
| **Address*:** | ÚOCHB<br>Flemingovo Náměstí 2<br>Praha<br>166 10 |
| **Sample type:** | DNA isolate |
| **Sample coll. method:** | by client |
| **Number of samples:** | 11 |

| Sample marking* | sampling date/passage* | receive date | received by |
|---|---|---|---|
| **FTSEC190** | N/A | 24.08.2022 | Hod |

**Analysis date:** 24.08.2022 - 02.09.2022

**Used methods:** SOP_T1_014 Autentizace_buněčných_linií (STR profil)_fragmentační_analýza_DNA

**Test result:**
Cell line marked FTSEC190 is identical to reference cell line hTERT FT 190 (CRL-3444)
No. of mismatched alleles 0. Conformity level is 100 %.
According to ICLAC guide, cell line is authenticated when conformity level is more than 80 %.

The test results are applicable to tested samples only (in the condition as delivered).

* information from the contracting authority, Testing laboratory is not responsible for this information

**Report date:** 02.09.2022

| **Person responsible for the test** | **Report released by** |
|---|---|
| Mgr. Dagmar Hodíková<br>Deputy head of Testing laboratory | Mgr. Veronika Šmídová<br>Head of Testing laboratory |
| Mgr. Dagmar Hodíková — Digitálně podepsal Mgr. Dagmar Hodíková Datum: 2022.09.20 10:23:13 +02'00' | Mgr. Veronika Šmídová — Digitálně podepsal Mgr. Veronika Šmídová Datum: 2022.09.20 12:16:24 +02'00' |

| Polymorfism | Sample* | | | Reference cell line | | | Degree of compliance | |
|---|---|---|---|---|---|---|---|---|
| | FTSEC190 | | | hTERT FT 190 CRL-3444 | | | | |
| Amelogenin | X | X | | X | X | | + | + |
| CSF1PO | 10 | 12 | | 10 | 12 | | + | + |
| D13S317 | 11 | 13 | | 11 | 13 | | + | + |
| D16S539 | 9 | 10 | | 9 | 10 | | + | + |
| D18S51 | 12 | 16 | | 0 | 0 | | N/A | N/A |
| D19S433 | 14 | 14 | | 0 | 0 | | N/A | N/A |
| D21S11 | 30 | 30 | | 0 | 0 | | N/A | N/A |
| D2S1338 | 17 | 18 | | 0 | 0 | | N/A | N/A |
| D3S1358 | 14 | 15 | | 0 | 0 | | N/A | N/A |
| D5S818 | 11 | 11 | | 11 | 11 | | + | + |
| D7S820 | 11 | 12 | | 11 | 12 | | + | + |
| D8S1179 | 8 | 14 | | 0 | 0 | | N/A | N/A |
| FGA | 22 | 27 | | 0 | 0 | | N/A | N/A |
| Penta D | 0 | 0 | | 0 | 0 | | N/A | N/A |
| Penta E | 0 | 0 | | 0 | 0 | | N/A | N/A |
| TH01 | 7 | 7 | | 7 | 7 | | + | + |
| TPOX | 8 | 8 | | 8 | 8 | | + | + |
| vWA | 16 | 17 | | 16 | 17 | | + | + |

No. of mismatches: 0
Conformity level: 100%

Used database: ATCC

* information from the contracting authority, Testing laboratory is not responsible for this information

Report no. 5225-9/2022

## OVCAR-3

| Allelic Marker | Lorenzi et. al* | NCI60 | Lot # 502297-- 8.19.2022 | Comments |
|---|---|---|---|---|
| D8S1179 | 10,15 | 10,15 | 10,15 | |
| | | | | |
| D21S11 | 29,31.2 | 29,31.2 | 29,31.2 | |
| | | | | |
| D7S820 | 10,10 | 10,10 | 10,10 | SAMPLE MATCHES |
| | | | | 100% WITH THE |
| CSF1PO | 11,12 | 11,12 | 11,12 | NCI60 SCREEN |
| | | | | |
| D3S1358 | 17,18 | 17,18 | 17,18 | |
| | | | | |
| TH01 | 9,9 | 9,9 | 9,9 | |
| | | | | |
| D13S317 | 12,12 | 12,12 | 12,12 | |
| | | | | |
| D16S539 | 12,12 | 12,12 | 12,12 | |
| | | | | |
| D2S1338 | 17,21 | 17,21 | 17,21 | |
| | | | | |
| D19S433 | 16.2,16.2 | 14,16.2 | 14,16.2 | |
| | | | | |
| vWA | 17,17 | 17,17 | 17,17 | |
| | | | | |
| TPOX | 8,8 | 8,8 | 8,8 | |
| | | | | |
| D18S51 | 13,13 | 13,13 | 13,13 | |
| | | | | |
| AMEL | X,X | X,X | X,X | |
| | | | | |
| D5S818 | 11,12 | 11,12 | 11,12 | |
| | | | | |
| FGA | 21,21 | 21,21 | 21,21 | |
| | | | | |

* = Lorenzi PL et al. DNA fingerprinting of the NCI-60 cell line panel. *Mol. Cancer Ther* 2009;8(4) pp 713-24.

# OVCAR-4

| Allelic Marker | Lorenzi et. al* | NCI60 | Lot # 507325--8.19.2022 | Comments |
|---|---|---|---|---|
| D8S1179 | 13,13 | 13,13 | 13,13 | |
| | | | | |
| D21S11 | 28,31 | 28,31 | 28,31 | |
| | | | | |
| D7S820 | 10,11 | 10,11 | 10,11 | SAMPLE MATCHES |
| | | | | 100% WITH THE |
| CSF1PO | 10,10 | 10,10 | 10,10 | NCI60 SCREEN |
| | | | | |
| D3S1358 | 15,15 | 15,15 | 15,15 | |
| | | | | |
| TH01 | 9,9 | 9,9 | 9,9 | |
| | | | | |
| D13S317 | 9,9 | 9,9 | 9,9 | |
| | | | | |
| D16S539 | 11,11 | 11,11 | 11,11 | |
| | | | | |
| D2S1338 | 23,23 | 23,23 | 23,23 | |
| | | | | |
| D19S433 | 13,15 | 13,15 | 13,15 | |
| | | | | |
| vWA | 14,18 | 14,18 | 14,18 | |
| | | | | |
| TPOX | 8,8 | 8,8 | 8,8 | |
| | | | | |
| D18S51 | 15,15 | 15,15 | 15,15 | |
| | | | | |
| AMEL | X,X | X,X | X,X | |
| | | | | |
| D5S818 | 13,13 | 13,13 | 13,13 | |
| | | | | |
| FGA | 21,21 | 21,21 | 21,21 | |
| | | | | |

* = Lorenzi PL et al. DNA fingerprinting of the NCI-60 cell line panel. *Mol. Cancer Ther* 2009;8(4) pp 713-24.
